# Different metazoan parasites, different transcriptomic responses, with new insights on parasitic castration by digenetic trematodes in the schistosome vector snail *Biomphalaria glabrata*

**DOI:** 10.1186/s12864-024-10454-4

**Published:** 2024-06-17

**Authors:** Lijun Lu, Lijing Bu, Martina R. Laidemitt, Si-Ming Zhang, Eric S. Loker

**Affiliations:** Department of Biology, Center for Evolutionary & Theoretical Immunology, Parasite Division, Museum of Southwestern Biology, University of New Mexico, Albuquerque, 87131 USA

**Keywords:** *Biomphalaria glabrata*, *Schistosoma mansoni*, *Echinostoma paraensei*, *Daubaylia potomaca*, Transcriptomics, RNA-Seq, Parasitic castration, Fecundity compensation

## Abstract

**Background:**

Gastropods of the genus *Biomphalaria* (Family Planorbidae) are exploited as vectors by *Schistosoma mansoni*, the most common causative agent of human intestinal schistosomiasis. Using improved genomic resources, overviews of how *Biomphalaria* responds to *S. mansoni* and other metazoan parasites can provide unique insights into the reproductive, immune, and other systems of invertebrate hosts, and their responses to parasite challenges.

**Results:**

Using Illumina-based RNA-Seq, we compared the responses of iM line *B. glabrata* at 2, 8, and 40 days post-infection (dpi) to single infections with *S. mansoni*, *Echinostoma paraensei* (both digenetic trematodes) or *Daubaylia potomaca* (a nematode parasite of planorbid snails). Responses were compared to unexposed time-matched control snails. We observed: (1) each parasite provoked a distinctive response with a predominance of down-regulated snail genes at all time points following exposure to either trematode, and of up-regulated genes at 8 and especially 40dpi following nematode exposure; (2) At 2 and 8dpi with either trematode, several snail genes associated with gametogenesis (particularly spermatogenesis) were down-regulated. Regarding the phenomenon of trematode-mediated parasitic castration in molluscs, we define for the first time a complement of host genes that are targeted, as early as 2dpi when trematode larvae are still small; (3) Differential gene expression of snails with trematode infection at 40dpi, when snails were shedding cercariae, was unexpectedly modest and revealed down-regulation of genes involved in the production of egg mass proteins and peptide processing; and (4) surprisingly, *D. potomaca* provoked up-regulation at 40dpi of many of the reproduction-related snail genes noted to be down-regulated at 2 and 8dpi following trematode infection. Happening at a time when *B. glabrata* began to succumb to *D. potomaca*, we hypothesize this response represents an unexpected form of fecundity compensation. We also document expression patterns for other *Biomphalaria* gene families, including fibrinogen domain-containing proteins (FReDs), C-type lectins, G-protein coupled receptors, biomphalysins, and protease and protease inhibitors.

**Conclusions:**

Our study is relevant in identifying several genes involved in reproduction that are targeted by parasites in the vector snail *B. glabrata* and that might be amenable to manipulation to minimize their ability to serve as vectors of schistosomes.

**Supplementary Information:**

The online version contains supplementary material available at 10.1186/s12864-024-10454-4.

## Background

Members of the phylum Mollusca, particularly including the speciose class Gastropoda, support a wide range of parasites, among them several types of metazoan parasites, including digenetic trematodes, parasitic copepods, nematodes, insects, and even other molluscs [1–3]. These different lineages of metazoan parasites, with very different life cycles and phylogenetic backgrounds, can be thought of as probes to elicit different responses from their gastropod hosts, thereby illuminating the extent and range of molluscan physiological responses, including both immune and reproductive responses.

Towards this end, building on much improved genomic resources available for the iM line (inbred M line) of *Biomphalaria glabrata* (family Planorbidae) [4, 5], we have sought to characterize the responses of *B. glabrata* to three different relevant metazoan parasites. Two are digenetic trematodes (Digenea), the first being *Schistosoma mansoni*, the causative agent of human intestinal schistosomiasis. *S. mansoni* falls within one of two well-supported orders within the Digenea, the Diplostomida [6], and exemplifies a life cycle pattern in which parasite eggs passed in human feces hatch in freshwater, each releasing a free-swimming miracidium that penetrates its intermediate host, a snail like *B. glabrata*, and transforms into a mother sporocyst which undergoes asexual reproduction to produce sac-like daughter sporocysts. These in turn produce multitudes of fork-tailed cercariae that are released into the water. Cercariae penetrate the skin of their human definitive hosts and will develop intravascularly into sexually reproducing adult worms, thereby completing their life cycle.

The second digenean, *Echinostoma paraensei*, represents the Order Plagiorchiida [6]. The *E. paraensei* life cycle involves rediae rather than just sporocysts as intramolluscan stages [7]. Unlike sac-like sporocysts, rediae have a mouth surrounded by an oral sucker and a blind gut of varying length. In this case, an egg passed in the feces of a rodent host hatches to release a miracidium that penetrates *B. glabrata* and moves to the pericardium where it develops into a sac-like sporocyst which produces a generation of mother rediae that disseminate from the heart to other organs. These produce daughter rediae which then produce cercariae which are released from the snail. The cercariae encyst in another snail as metacercariae which if ingested by a rodent definitive host will develop into sexually reproducing adult worms that produce eggs. Whereas *S. mansoni* is almost exclusively a parasite of certain species of *Biomphalaria*, *E. paraensei* can develop in both *Biomphalaria* species and in *Physa rivalis*, the latter representing a different basommatophoran family, the Physidae [8].

The third metazoan we include is a nematode, *Daubaylia potomaca* (family Cephalobidae), that is unusual for being exclusively parasitic in planorbid snails [9, 10]. Gravid or inseminated female worms penetrate *B. glabrata* to initiate infections. They produce eggs which hatch releasing L3 larvae which undergo two molts to become adults. Multiple cycles of reproduction can occur in the snails and, in *B. glabrata*, a short period prior to the snails dying, gravid or inseminated females emerge from the snail and initiate infection in another snail.

Investigations to ascertain the nature of gastropod responses to infection, particularly after exposure of *B. glabrata* to the medically relevant *S. mansoni* have increased in number and sophistication in recent years, taking advantage of greatly improved genomics resources beginning with the initial BB02 *B. glabrata* genome project [11], followed by improved genomes based on homozygous lines of *B. glabrata* either resistant (iBS90) or susceptible (iM line) to *S. mansoni* [4], achieving chromosome level resolution in the latter case. The chromosome-level assembly of the iM line genome is now accessible, featuring enhanced annotation and improved assembly [5]. Reference genomes for other species of *Biomphalaria* including *B. straminea* [12], *B. pfeifferi* [13] and *B. sudanica* [14] and for *Bulinus truncatus* [15], a snail host for *S. haematobium*, the agent of urinary schistosomiasis, are now all available. Several sequence-based technologies have also been employed to further our understanding of *Biomphalaria* genomics including microarrays [16], transcriptomics [17–19], bioinformatics studies of non-coding RNAs [20] and whole-genome association studies [21], all topics covered more extensively in pertinent reviews [22–24].

Previous studies have also specifically highlighted differences in the responses of *B. glabrata* to different categories of pathogens or parasites including bacteria and digenetic trematodes. In a qPCR-based study, Hertel et al. [25] noted that the responses of particular *B. glabrata FREP* genes to *S. mansoni* and *E. paraensei* differed and suggested the two trematodes provoked fundamentally different kinds of responses in the snail host. Microarray studies discerned that the two trematodes provoked predominantly down-regulated responses over most of the course of a 32 day observation period [26]. An RNA-Seq study of *Physella acuta* suggested this snail can alter its defense responses from a focus on trematode infection to one more directed to opportunistic pathogens later during the course of infection [27]. Other studies have emphasized that *B. glabrata* activates different biomphalysin genes depending on the particular pathogen or parasite to which it is exposed [19, 28–30]. Even among different trematode species colonizing the same snail species, distinctive host responses might be expected given that the different trematode species involved have their own characteristic microbiomes [31]. In contrast, some evidence points to similarities among proteases from snail-infective nematodes and trematode larvae, suggestive of the possibility that successful parasite infective strategies might be limited, possibly limiting the range of host responses as well [32]. To this date, none of the available next-gen expression studies provide a comprehensive, genome-based study of a comparison of gastropod responses to metazoan parasites representing different phyla.

In the present study, we use RNA-Seq to provide a more unbiased view of how iM line *B. glabrata* [5], responds to the digeneans *S. mansoni* or *E. paraensei*, or to the nematode *D. potomaca*, at representative times during their intramolluscan development. The iM line chromosome-level assembly is improved from scaffold-level assemblies in having enhanced annotation and improved assembly and serves as the reference genome for genome mapping and RNA-Seq analysis for this study. As this study encompasses the use of both juvenile and adult snails, it can also provide insights into how infection influences the process of sexual maturation and parasite-induced castration, the latter a phenomenon well-known to occur in molluscs following exposure to trematode infection [33].

## Methods

### Snails and parasites used in this study

M line snails used in this study were from the inbred homozygous M line (iM line) [4], established in our laboratory since 2001. The iM line was selected to increase genetic uniformity and diminish the variability that has impeded studies of snail biology in the past, and to provide a more stable platform for developing snail control strategies. The iM line snails used in this study were derived from the 72nd generation of inbreeding, and were reared and maintained using typical methods for snail lab colonies [34]. The recently published iM line genome was based on snails from the 81st generation of selfing [5].

The PR-1 strain of *Schistosoma mansoni* and the echinostomatid digenean *Echinostoma paraensei* were maintained at the Biology Department of the University of New Mexico as previously described [34, 35]. *Daubaylia potomaca* is one of 7–8 species of this genus of snail-infecting nematodes (Nematoda: Cephalobidae) [36]. Mature *D. potomaca* females either inseminated or gravid are the infective stages for snails of the family Planorbidae [10, 37]. The *D. potomaca* used in this study were freshly-isolated from *Planorbella* (*Helisoma*) *duryi* obtained from a local pond at Shady Lakes (35°13’03.5"N, 106°35’48.3"W) in Albuquerque, New Mexico. As demonstrated during this study, iM line *B. glabrata* permit full larval development of, that is they are compatible with, all three parasites we used.

### Experimental infections and sample collection

To better understand interactions between iM line *B. glabrata* and these three metazoan parasites in a less biased way at the gene expression level, the following transcriptomic study was set up. Juvenile snails (5–8 mm) were individually put in the wells of 24-well plates, in 2 ml artificial spring water (ASW) and exposed to freshly-hatched or isolated parasites: ~10 PR-1 *S. mansoni* or ~ 10 *E. paraensei* miracidia for 6 h, or 5 ~ 7 *D. potomaca* females for 16–20 h. Control snails were treated similarly but were not exposed to any parasites. Snails of the four groups (controls, or those exposed to one of the three parasites) were moved to separate aerated aquaria containing ASW at 25–27 °C, on a 12:12 h light-dark cycle, and fed with lettuce *ad libitum*.

As summarized in Table 1, snails exposed to each parasite were sampled at random from their respective aquaria at 2, 8, or 40 days post-infection (dpi). Unexposed control snails were sampled at 2dpi and also served as controls for the snails with 8 day infections, as controls at these two times did not differ in size, had not laid eggs and were considered juveniles. By 40dpi, both parasite-exposed and control snails were considered as adults because the control snails had produced eggs. The unexposed adult control snails were the only snails that produced egg masses during this study (eggs numerous but not counted).

**Table 1:** Study design for iM line snails used for RNA-Seq. All snails were juveniles (5–8 mm shell diameter) at the start of this study. All parasite infections were administered on the same day. Three snails were sequenced for each of the indicated combinations of parasite species vs. days-post infection. Snails infected for 2 or 8dpi were juveniles. By 40dpi, parasite-infected snails were of adult size (> 10 mm shell diameter). Additionally, 3 juvenile and 3 adult control snails, treated identically to parasite-infected snails except they were not exposed to parasites, were sampled at the indicated times. In total, 33 snails were subjected individually to RNA-Seq

Snails Exposed To:			Days Post-Infection (dpi)		
			2	8	40
*Schistosoma mansoni*			3	3	3
*Echinostoma paraensei*			3	3	3
*Daubaylia potomaca*			3	3	3
Unexposed control snails			3		3*

*Were observed to produce eggs confirming their status as adults

**Fig. 1:**
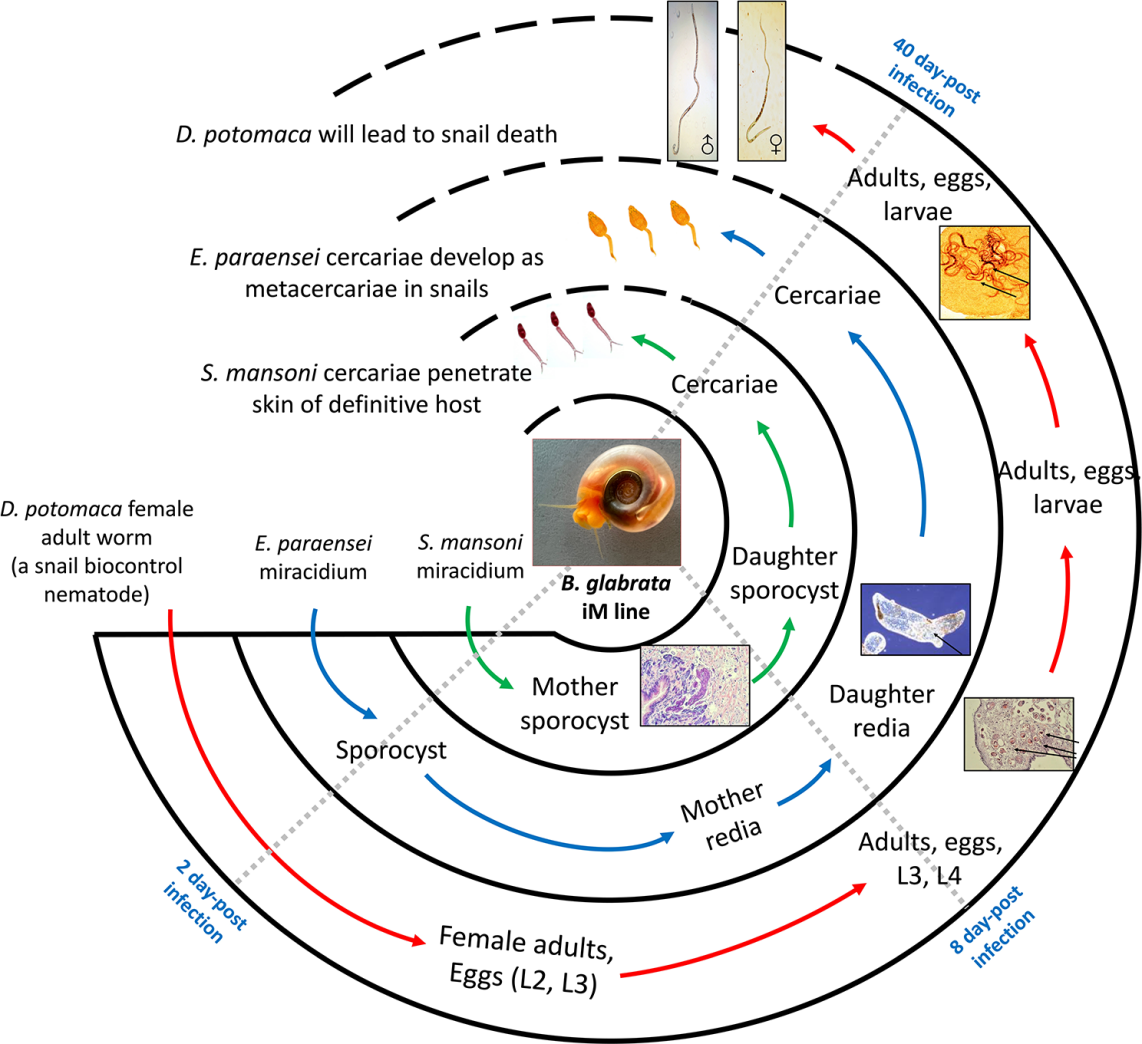
Life cycles and illustrations of the different developmental stages of *S. mansoni*, *E. paraensei* and *D. potomaca* in iM line *B. glabrata*. Shown for each parasite species used is an intramolluscan life cycle with different life cycle stages connected by arrows, and the approximate times post-infection indicated (2, 8 and 40 days post-infection). Presence of developing parasites of the expected species was confirmed in all parasite-exposed species (see text)

The sampling times were chosen to match key stages of each parasite development in iM line snails (Fig. 1). For *S. mansoni* (green arrow illustration), 2 days, early infection and mother sporocyst establishment; 8 days, daughter sporocyst production; 40 days, full-fledged infection with daughter sporocysts production and release (shedding) of cercariae. For *E. paraensei* (blue arrow), 2 days, sporocyst establishment; 8 days, mother/daughter redia production; 40 days, full-fledged infection with daughter rediae production and release of cercariae. For *D. potomaca* (red arrow), 2 days, females present with eggs containing L2 and L3 larvae; 8 days, adults, eggs and L3 stages and L4 stages present; 40 days, eggs, larvae and adult nematodes are present, and emergence of adult females normally presages the death of the host snail, which typically occurs between 26 and 44dpi [9]. All our *D. potomaca*-infected snails were alive at the time sampled. Note that for the *E. paraensei* exposed snails, intracardiac sporocysts can be seen at 2dpi [34], so only those snails visibly harboring sporocysts were kept for the experiment. As indicated in Table 1, at both 2 and 8dpi, 3 snails were selected from each of the three groups exposed to a different parasite species for further processing. At 40dpi, all remaining snails of *S. mansoni* or *E. paraensei* exposed groups were isolated and checked for cercariae shedding. For this purpose, snails were placed individually in wells of 12-well plates with 2 ml ASW and held under artificial light for 2 h, only shedding snails were sampled for extraction and sequencing. For the *D. potomaca* exposed group at 40dpi, all remaining snails were cracked to confirm *D. potomaca* infection immediately prior to TRIzol preservation.

Snails sampled for this study were individually preserved in TRIzol reagent (Invitrogen) and stored at -80 °C until extraction. The RNA extraction process followed TRIzol manufacturer’s instructions (Invitrogen) with modification of a few steps to yield more RNA [38]. RNA samples were further purified using the PureLink RNA Mini Kit (Thermo Fisher Scientific) to yield high-quality RNA. Quality and quantity of RNA extracted from each sample were measured with a Qubit RNA HS (High Sensitivity) Assay kit and Agilent 2100 Bioanalyzer (Agilent RNA 6000 Pico kit), respectively. The RNAs used in this RNA-Seq study all meet the requirements: > 1 µg total RNA and RIN (RNA Integrity Number) > 7.0. RNA samples were stored at -80 °C and were used for library preparation within one week. To ensure the experimental snails used for sequencing were truly infected with *S. mansoni*, *E. paraensei*, or *D. potomaca*, conventional PCR (cPCR) assays were applied for each group with the extracted gDNA. The parasite specific primers for *S. mansoni* [39], *E. paraensei* [40], or *D. potomaca* [36, 41] were used to confirm parasite infection status for each parasite-exposed snail (Table 2). Only those snails confirmed to be positive with cPCR, or that had shed cercariae (*S. mansoni*, or *E. paraensei* at 40d), or were confirmed positive by dissection (*D. potomaca* at 40d) were considered to be “infected” snails and readied for sequencing.

**Table 2:** Primers used to confirm parasite infection in the parasite-infected snails

Target parasite	Target gene	Primer sequence	Amplicon size	Reference
*S. mansoni*	*ND5*	F: 5’-ATTAGAGGCAATGCGTGCTC-3’	302 bp	[39]
		R: 5’-ATTGAACCAACCCCAAATCA-3’		
*E. paraensei*	*ND1*	F: 5’-AGATTCGTAAGGGGCCTAATA-3’	530 bp	[40]
		R: 5’-ACCACTAACTAATTCACTTTC-3’		
*D. potomaca*	*18 S rRNA*	F: 5’- CGCGAATRGCTCATTACAACAGC-3’	900 bp	[41]
		R: 5’- GGGCGGTATCTGATCGCC-3’		
	*28 S rRNA*	F: 5’- AGCGGAGGAAAAGAAACTAA-3’	1,000 bp	[36]
		R: 5’- TCGGAAGGAACCAGCTACTA-3’		

### Library preparation, RNA-sequencing and differential expression (DE) analysis

Three replicates per group per sampling time point were selected for library preparation (overview of snail groups and biological replicates) with a total of 33 snails sampled (Table 1). Complementary DNA (cDNA) synthesis and Illumina NextSeq 500 sequencing was performed at the Molecular Biology Facility, Biology Department, the University of New Mexico. Synthesis of cDNA from each sample and library preparation followed the KAPA mRNA HyperPrep Kit Illumina® Platforms protocol (Roche). Complementary DNA libraries were paired-end sequenced (2 × 150 base reads) on an Illumina NextSeq 500 instrument (Illumina).

Based on the sequencing quality, raw reads were trimmed and filtered using Trimmomatic v0.36 [42] with a slide window of 4 nt, average score above 20 and minimum length of 36 nt. Filtered high quality reads from each parasite-exposed group were sorted based on the sample-specific adapters and mapped to the annotation-updated *B. glabrata* iM line strain genome [4] using STAR 2.5.3a [43]. The trimmed “clean” reads of each parasite-exposed individual were mapped separately to the corresponding parasite reference genome: *S. mansoni* (SM_V10, 2022-11-WormBase) [44, 45], or *Echinostoma caproni* (GCA_900618425.1), respectively. Reads in each replicate shared between snail and parasite were regularly at or under 1% of the total sequencing reads in this study (Table S1). Due to the low percentage of shared reads observed and the unknown impact of removing them, we kept the shared reads for mapping to the *B. glabrata* iM line genome for the DE analysis (Table S1). Clean reads (~ 11 million unique and shared snail reads/snail sample) with a quality score above 20 and length of at least 36 nt (post-trimming) were mapped to the *B. glabrata* iM line reference genome for differential expression analysis. For the *D. potomaca* exposed group, considering the unavailability of a reference genome from a closely related species, we estimated the proportion of *D. potomaca* reads using remaining sequencing reads that did not map to the iM line genome.

Gene expression levels were estimated using the software featureCounts [46]. Differential gene expression (DE) analysis was performed by using EBSeq v1.22.0 [47] with normalized clean reads. To identify a list of differentially expressed (DE) genes with a false discovery rate (FDR) controlled at α when comparing two biological conditions, the genes with a posterior probability of being DE (PPDE) higher than (1 – α) were selected as DE. The default statistic cut-off value of PPDE in EBSeq was ≥ 0.95 and considered as differentially expressed genes. DE analysis results were organized using SARTools 1.6.6 [48]. Workflow of read trimming and mapping was built using Unix shell commands with application GNU-Parallel [49] to perform jobs in parallel. Due to the large number of DE genes discovered, only those DE genes with fold change (FC) value greater than 2 (FC ≥ 2) in either up- or down- regulated were taken into further analyses. Within each parasite-exposed group, values for parasite-exposed snails were compared to baselines from unexposed control snails time-matched to the same exposed group (Table 1). More detailed description of the library preparation, sequencing and DE analysis can be found in Lu et al. [50, 51].

### Other analyses

Signal peptide search was performed using SignalP 4.0 [52]. Table summary and figure generation were performed with the R statistical computing environment [53] and Bioconductor [54], including the following packages: ggplot2 [55], reshape2 [56], magic 2.0 (https://docs.ropensci.org/magick/index.html), ggtree 3.9 [57], and openxlsx v4.1.0.1 [58]. Intermediate data analysis was done with an in-house parallel pipeline facilitated with the GNU Parallel tool [49]. On the KEGG (Kyoto Encyclopedia of Genes and Genomes) [59] website https://www.kegg.jp/, we used the BlastKOALA (BLAST-based KO annotation and KEGG mapping) [60] tool to investigate the pathways relevant to this study. We followed the protocol to upload the amino acid sequences of the iM line *B. glabrata* [5] and concentrated on identifying meiosis-related pathways.

Many reproduction-related genes were found by searching the lists of differentially expressed (DE) genes by the following general terms: reproduction; meiosis; meiotic; synapto-; sperm; gonad; ovu-; egg; yolk; cell division; centromere; centrosome-; testis; vitello-; perivit-; apolipo-; kineto-; adrenergic; neuro-; prostatic; galac-; beta-galac-; corazonin; caudal; hormone; caudodorsal; GnRH; ferritin. Others were found by manually going through the lists.

## Results

### Overview of sequencing and raw reads obtained

A total of 385 million paired-end reads were generated from the 33 snail samples indicated in Table 1. Presence of the expected parasite in each exposed snail selected for further study was confirmed as described in the materials and methods by cPCR, by the presence of parasite reads, or by the visible appearance of parasites upon shedding or dissection (Table S2, S3). A principal components analysis (PCA) plot showing an overview of the expression profiles for all 33 snails is shown in Fig S1.

### An overview of differential expression (DE) of iM line genes exposed to 3 different parasites, at 2, 8 and 40dpi

**Fig. 2:**
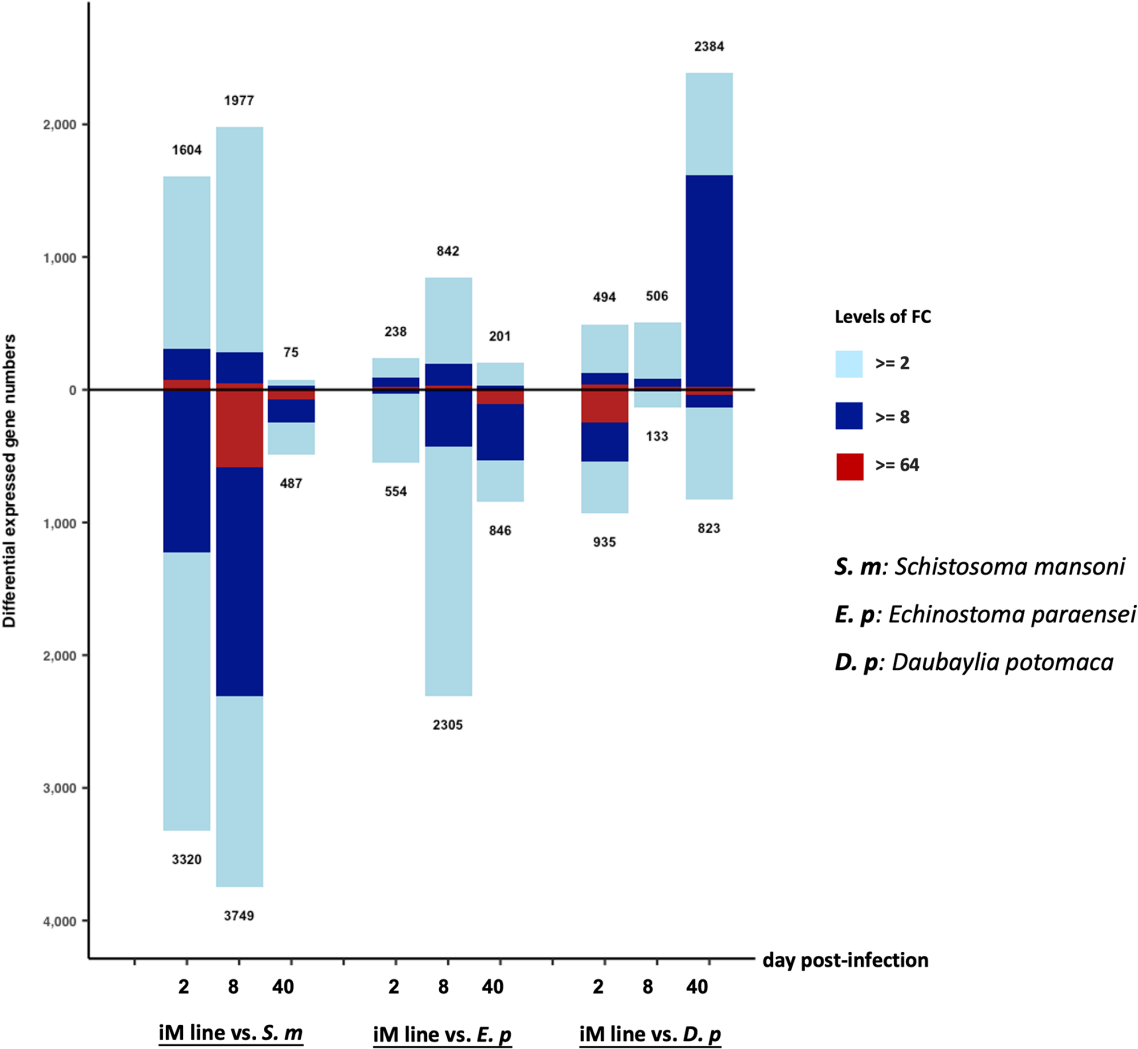
Bar graph showing all DE genes in the three snail-parasite exposure groups at 2, 8 and 40 days post infection (dpi). On the vertical axis, the number above the bar is the number of genes exhibiting an excess in transcripts in parasite-exposed snails relative to time-matched unexposed control snails. The number below the bar is the number of genes with an excess in transcripts for control snails relative to parasite-exposed snails. The three colors in each bar indicate fold changes (FC) among comparisons: light blue 2–8; dark blue 8–64; and red greater than 64-fold

Using the criterion of a two-fold difference or greater, the total number of DE genes relative to controls identified for each of the 9 comparisons (3 time points for each of the 3 parasites) is shown in Fig. 2. For each parasite infected group, gene counts in the bar graph indicate DE genes with the posterior probability of differential expression (PPDE) ≥ 0.95 in the EBSeq DE analysis (Fig. 2). In interpreting this graph and the Venn diagrams to follow, the 2 and 8 day data are derived from snails that are juveniles (have yet to produce eggs) but were in the process of maturing, whereas snails from the 40d time point were of sufficient size and age to be judged adults, as also demonstrated by egg-laying of the 40d control snails. Our analysis is of whole-body samples, and this might lead to under-representation of some organs, like the relatively small brain, and over-representation of others, like the large head-foot, particularly for adult snails.

The overall DE patterns of the two trematode-exposed groups are similar in that down-regulated genes predominated at each time point, including those genes with the greatest changes in expression (from 8 to > 64-fold changes). The largest number of extreme expression changes were noted in *S. mansoni*-exposed snails at 8dpi. For both trematodes, it was surprising that the overall responses at 40d were relatively modest given the extent to which their bodies are occupied by trematode larvae. In contrast, for the *D. potomaca* exposed group, up-regulated features predominated at 8 and especially at 40dpi.

The responses of certain components of the basic transcription machinery in parasite-infected snails were examined to help understand the overall patterns observed (see Tables S4-S6 for details). This included subunits of DNA-directed RNA polymerase II, genes encoding zinc finger proteins (Table 3), and polyadenylate-binding protein 3. In general, the expression for these genes was down-regulated in snails with 2 or 8d infections with trematodes, and up-regulated in snails with 40d infections with *D. potomaca*. Some of the genes associated with basic aspects of snail energy metabolism showed a similar pattern. These included glycosyl hydrolase alpha-amylases, 6-phosphoglycerate kinase genes instrumental to ATP production during glycolysis, and several genes associated with ATP-dependent trans-membrane transfer (Table S7).

**Table 3:** Summary of expression of zinc finger-encoding genes (likely transcription-promoting factors) in snails infected for different time periods with the indicated parasite. For different times post-infection, relative to unexposed control snails, the number of up-regulated genes is indicated above the diagonal and the number of down-regulated genes below

Parasite	Day post-infection (dpi)		
	2	8	40
*S. mansoni*	12/54	15/56	0/1
*E. paraensei*	0/9	6/33	4/3
*D. potomaca*	5/7	1/3	37/2

### For each parasite, the number of DE snail genes unique to or in overlap among the three sampled time points (2, 8, and 40dpi) is indicated

**Fig. 3:**
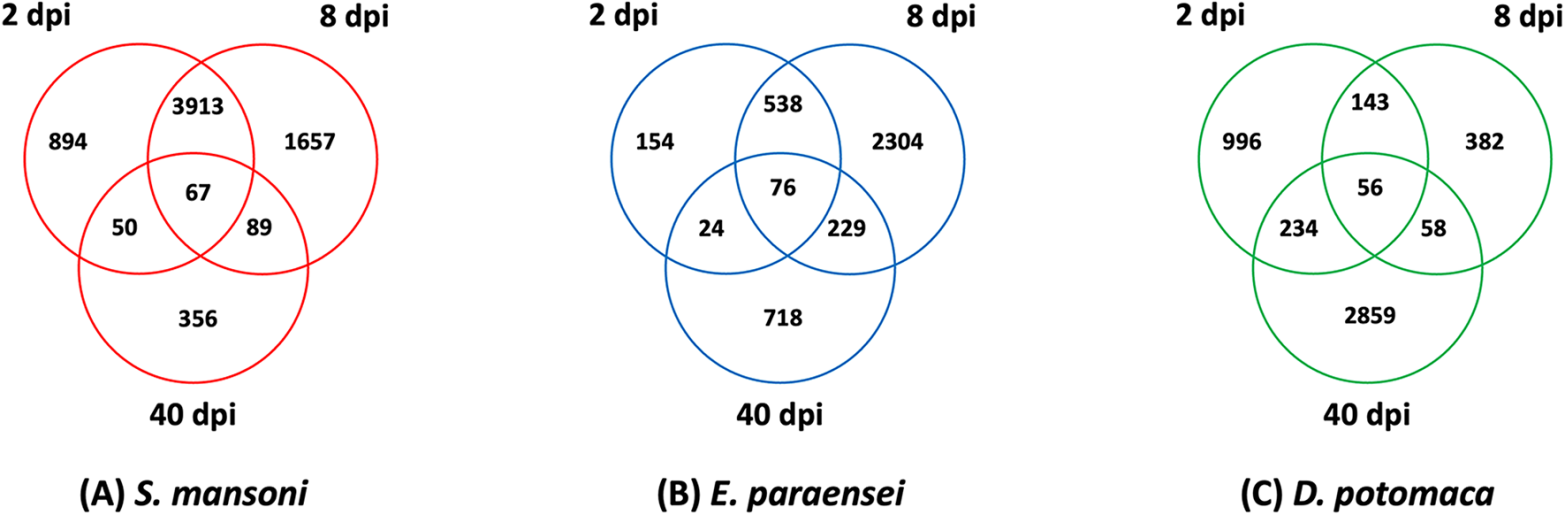
For each parasite, Venn diagrams indicating the number of DE snail genes unique to or in overlap among the three sampled time points (2, 8, and 40dpi)

In Fig. 3, for each parasite, the Venn diagrams indicate numbers of DE snail genes unique to or in overlap among the three sampled time points. Each diagram indicates large numbers of DE genes unique to each time point, suggestive of considerable temporal variation in snail responses to all three parasites. The 2 and 8dpi responses of snails exposed to *S. mansoni* show the greatest degree of overlap among the 3 diagrams; snails exposed to *E. paraensei* show a similar trend, but to a lesser extent. In general, the responses to *D. potomaca* exhibited more temporal specificity, with the 40dpi samples exhibiting not only a more extensive response but one marked by many distinctive features as compared to the two earlier time points (further details in Tables S8-S10).

### Gene-specific comparisons among the parasite-exposed snails at specific sampling

#### Time points

**Fig. 4:**
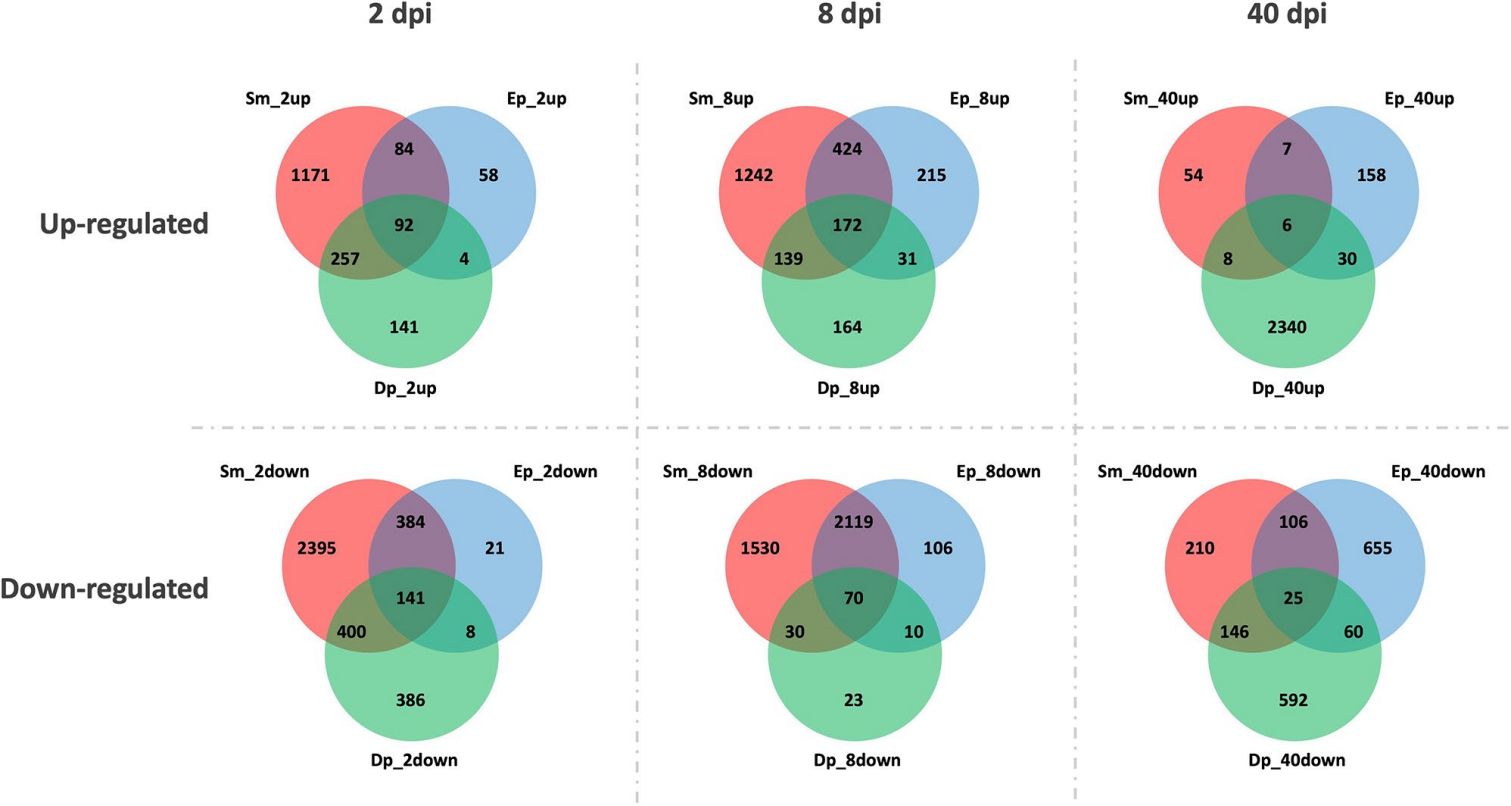
Venn diagram indicating for each time point the number of DE genes (up-regulated genes in the top row, down-regulated genes in the bottom row) unique to or in overlap among the 3 parasites

For each time point, the series of Venn diagrams in Fig. 4 indicate the number of DE genes expressed uniquely (up-regulated top row, down-regulated bottom row) to each parasite or in various combinations of overlap among the parasites. Many genes were uniquely expressed in response to *S. mansoni* at both 2 and 8dpi, and in response to *D. potomaca* at 40dpi. The highest instance of shared responses was between the two trematodes at 8dpi. For all three time points, the number DE genes in common to all 3 parasites was relatively modest by comparison to the overlapped responses engendered by the two trematode species (details in Tables S11-S13).

### Effects of the different parasites on expression patterns of genes related to *B. glabrata* reproduction (meiosis, gametogenesis, egg mass production)

**Fig. 5:**
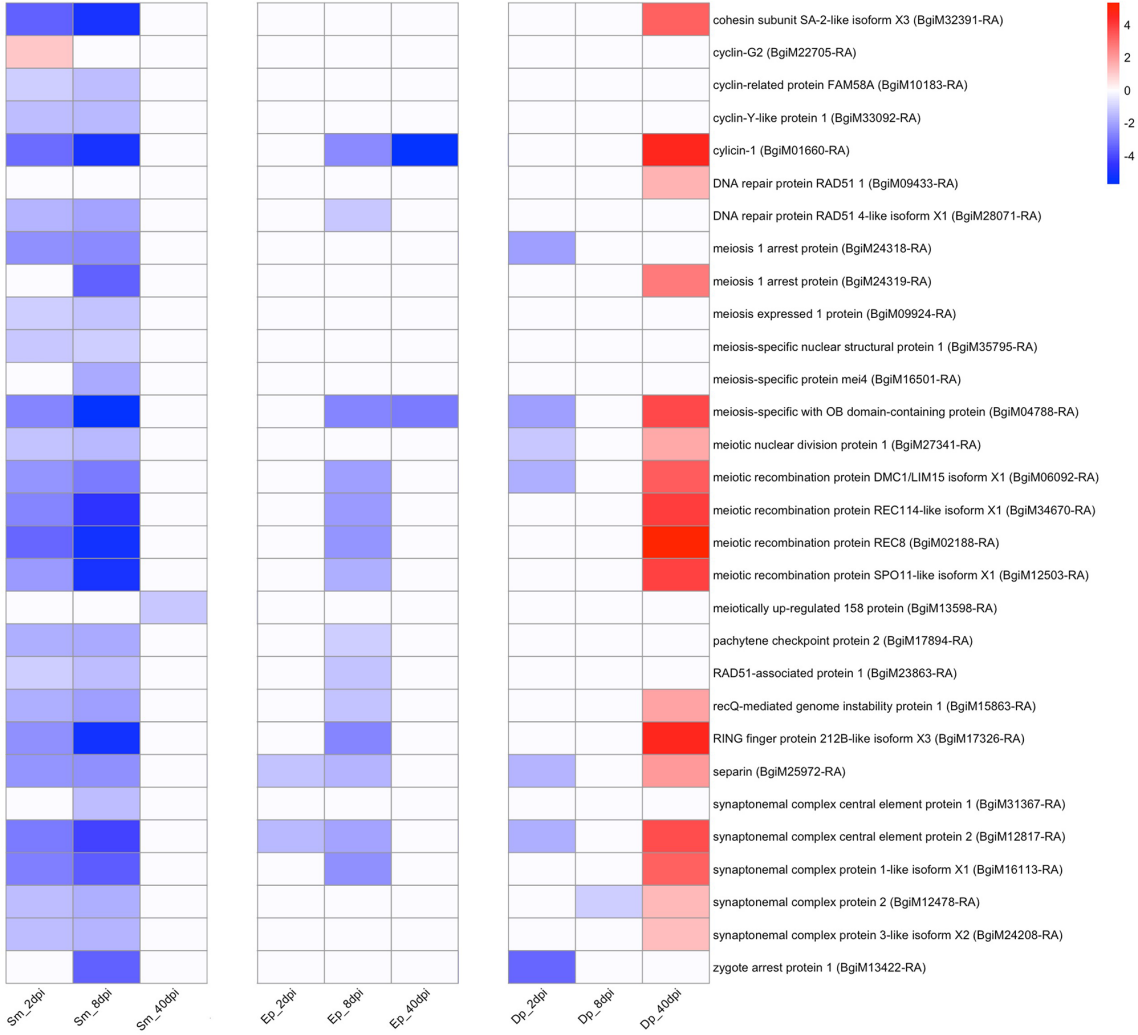
Heat map showing meiosis-associated genes with a predominant pattern of down-regulation in snails at 2 or 8dpi to *S. mansoni*, and to a lesser extent to *E. paraensei*. Note that some meiosis-related genes were also down-regulated in the early stages of *D. potomaca* infection, but then showed a surprising trend toward up-regulation at 40dpi

The most striking aspect of differential gene expression noted in this study was the down-regulation relative to unexposed control snails of many genes related to gametogenesis in juvenile snails infected for 2 or 8d with *S. mansoni*, and to a lesser extent with *E. paraensei*. Two-day infections with *D. potomaca* had a similar but a less prominent effect. This first came to light by searching DE genes by the term “meiosis”. Several meiosis-related transcripts were noted to be down-regulated (Fig. 5, Table S14).

This was initially surprising as the snails used for this study were “juveniles” at the time of parasite exposure, meaning they had yet to produce eggs. However, this does not necessarily mean they had yet to commence gonadogenesis and gametogenesis, processes that could be actively underway in juveniles of the size range used. It is noteworthy that for snails with 40d infections, no obvious down-regulation of meiosis-related genes was noted with trematode-infected snails, but the *D. potomaca*-infected snails exhibited up-regulation relative to the uninfected adult control snails. Other genes possibly associated with meiosis also showed a pattern similar to that seen in Fig. 5 (Table S14).

**Fig. 6:**
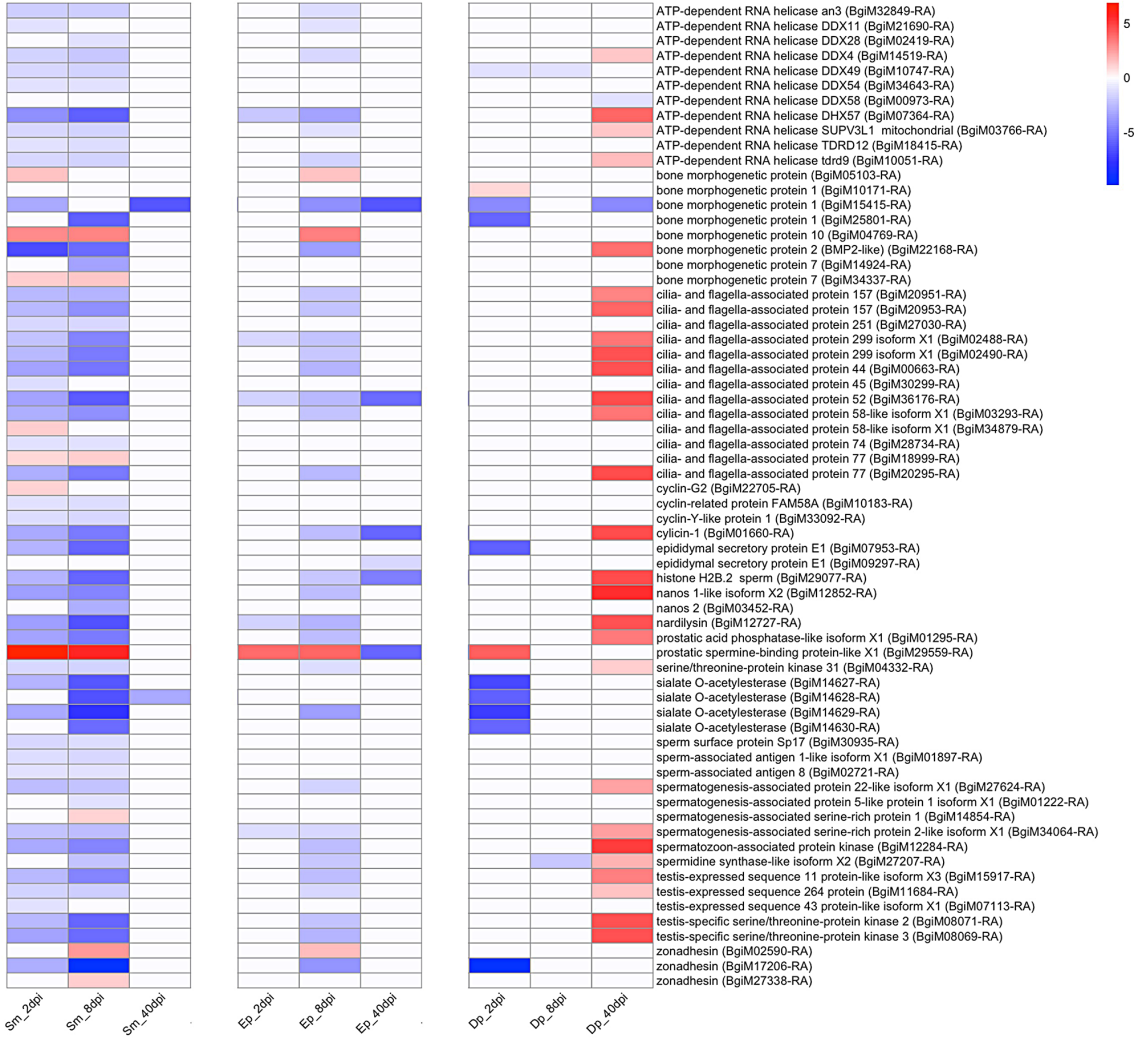
Heat map showing differential expression of a number of genes likely to be associated with spermatogenesis

Results of a KEGG pathway (Figure S2, Table S15) analysis focused on meiosis found that 20 of the 50 genes implicated in control of meiosis were down-regulated in snails exposed to one or the other trematode at 2 or 8 days, providing a confirmation that multiple genes related to meiosis were affected. Most of the down-regulated meiosis-related genes indicated in Figs. 5 and 6 are not included in the KEGG pathway, indicating an even greater aggregate impact on this process.

An overall pattern similar to that noted in Fig. 5 was again seen with respect to genes involved in spermatogenesis (Fig. 6, Table S16). In the developing ovotestis of juvenile snails, a strong early expression of genes involved in spermatogenesis might be expected because *B. glabrata* is a functionally protandrous species [61], and the number of cells involved in spermatogenesis is much greater than in oogenesis [62], so would be easier to detect.

It was again remarkable that 2 and 8d stages of *S. mansoni* (and again more modestly with 2 and especially 8d *E. paraensei* infections) development provoked mostly down-regulation across a spectrum of genes involved in spermatogenesis, relative to unexposed juvenile controls. Many of the genes on the list shown, by virtue of their names (their involvement with flagellar function, spermatogenesis or testis formation, for example) might be expected to affect sperm production. Other genes on the list have names less obviously associated with spermatogenesis but nonetheless are likely to play a role in this process (Fig. 6). For instance, *ATP-dependent RNA helicase*, otherwise known as the *vasa* gene, has been considered essential for germ cell development [63, 64] and has been used as a marker for germ cells. Similarly, *bone morphogenetic* proteins are implicated as growth factors, including for primordial germ cell development [65], and *sialate O-acetylesterases* often show testis-specific expression [66].

Genes associated with sperm tail structure, including axonemal dyneins and radial spoke proteins provide a good example of the extent to which spermatogenesis was affected in snails with 2 and 8d trematode infections. A total of 47 different axoneme-associated dynein-encoding genes and four radial spoke head genes (Table S17) were down-regulated. Both groups of genes show similar overall patterns of differential expression as noted above in Figs. 5 and 6. Several tubulin genes and *CAP-Gly domain-containing linker protein 1* involved in microtubule formation and elongation were also strongly down-regulated (Table S17). It is noteworthy that cytoplasmic dyneins associated with intracellular trafficking as opposed to sperm motility do not show the same trend towards down-regulation, suggesting that parasites target *axonemal dyneins* (Fig S3, Table S17).

Genes known from other molluscan studies to be involved in spermatogenesis [67] were also down-regulated (Table S18), again more extensively with *S. mansoni* than *E. paraensei*. The same was true for *tudor domain-containing protein 1*, involved in repressing transposon movement during gametogenesis. Additionally, genes associated with ovarian development in other molluscs were down-regulated in our snails at 2 or 8dpi with trematodes (Table S18), and other genes implicated in sexual differentiation [67] also showed a general pattern of down-regulation in our results (Table S18). Notable among the genes down-regulated were the major yolk proteins *vitellogenin-4* (by all 3 parasites at 2 and 8dpi), *vitellogenin A2* (by *E. paraensei* at 40dpi), and *yolk ferritin* (by *S. mansoni* at 8d and *D. potomaca* at 2d), the former two produced in the ovary and the latter in the digestive gland [68].

With respect to *D. potomaca*, the strong pattern of up-regulation of host reproduction genes at 40dpi was a surprise (Figs. 5 and 6, S3), as the snails involved were at a stage in the infection cycle when their prospects for further survival were limited. One of the remarkable aspects of the data is the extent to which the expression data for genes in snails with 2 or 8d trematode infections is a mirror image opposite of the data for snails with 40d *D. potomaca* infections.

**Fig. 7:**
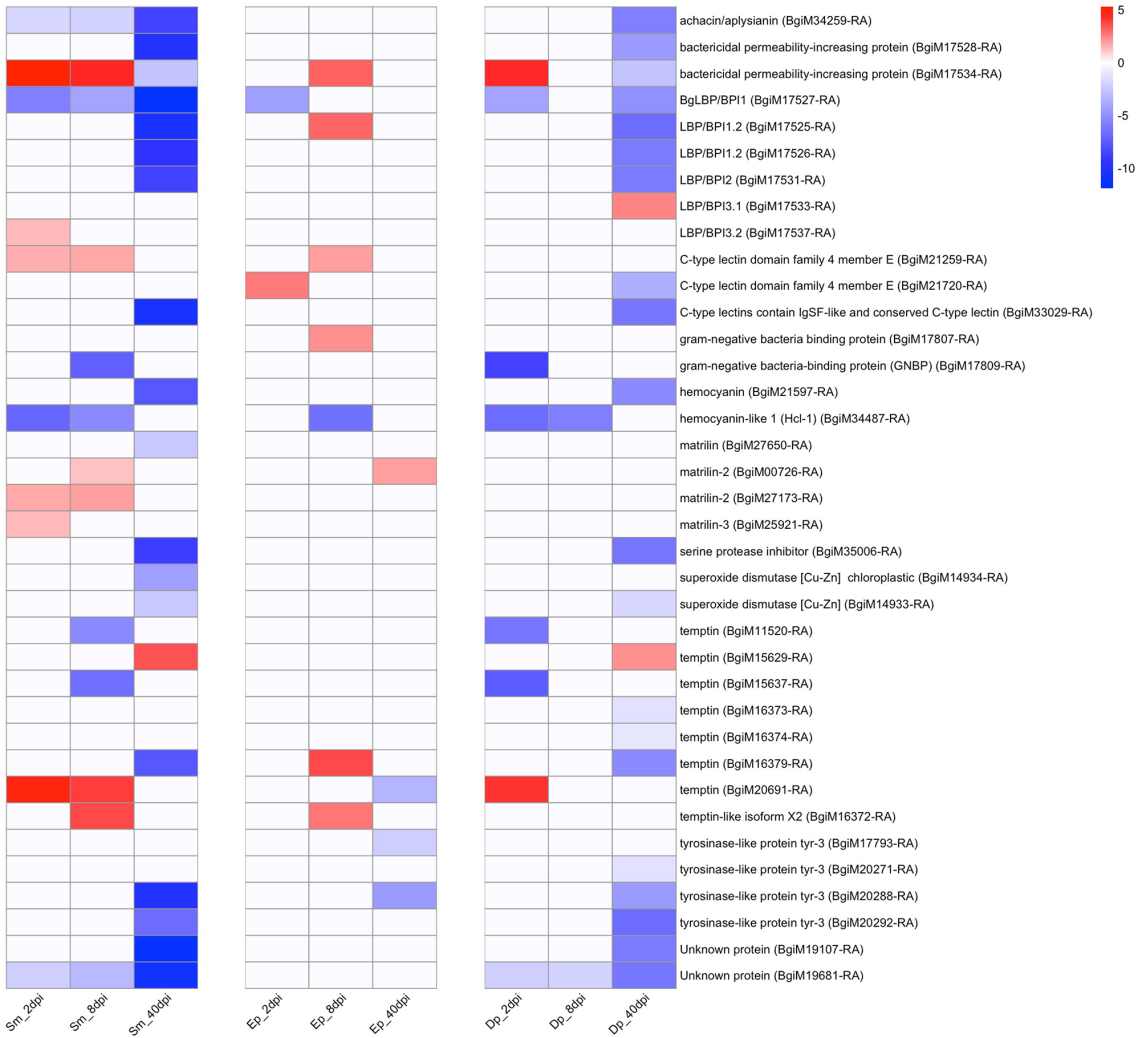
Heat map for genes encoding proteins in perivitelline and egg mass fluids

Whereas the reproduction-related processes identified so far relate to meiosis and gametogenesis, it is also well-appreciated that trematodes have a strong ability to affect female accessory reproductive organs like the albumen gland which produces the perivitelline fluid surrounding each fertilized egg, the pars contorta which produces two membrane around each egg, the muciparous gland which produces a secretion to hold the eggs together, and the oothecal gland which produce the capsule surrounding the egg mass [69]. Several proteins associated with egg and egg mass fluids have been identified in *B. glabrata* [70] and have guided our selection of genes in Fig. 7 (Table S19) we have used to monitor accessory female organ function.

The overall pattern noted for genes involved in egg and egg mass fluids is different from the previous heat maps because there is a predominance of down-regulated genes, many of them strongly so, for snails infected with *S. mansoni* for 40d. This was not surprising given existing literature on this topic indicating a sharp reduction in egg production for snails infected for this long, a time when cercarial production has commenced [71]. What was somewhat surprising is that snails with 40d *E. paraensei* infections showed a minimal effect on genes involved in egg mass protein production, even though their egg production was similarly impaired by a cercariae-producing infection. It is noteworthy, however, that the extent of the parasite burden in snails with 40d infections differed, with parasite reads counts for *S. mansoni* being on average over twice as high as what was recorded for *E. paraensei* (Table S1).

Also, whereas the pattern pertaining to gonadogenesis and gametogenesis gene expression seen previously for *D. potomaca* infections was contrary to what was noted for trematode infections, here the trend especially for 40d infections was very similar to that seen for *S. mansoni*, with many genes being down-regulated. Although many of the same genes were affected, the extent of down-regulation was usually not as strong in the *D. potomaca*-infected as in the *S. mansoni*-infected snails.

Among the genes that were strongly down-regulated in snails with 40d *E. paraensei* infections (Fig S4, Table S20), *endothelin-converting enzyme-like 1* was most noteworthy. Activity for this enzyme, generally considered to be a neuropeptide processing enzyme [72], has been localized to the reproductive organs in some invertebrates [73]. Other genes strongly down-regulated in snails with 40d *E. paraensei* infections were *thyrotropin-releasing hormone-degrading ectoenzyme, membrane metallo-endopeptidase-like 1, aminopeptidase N*, and *serine carboxypeptidase CPVL*, all genes encoding proteins with peptide cleavage activity.

### Genes associated with neural development or maintenance, neuro-endocrine communication and extracellular signaling

**Fig. 8:**
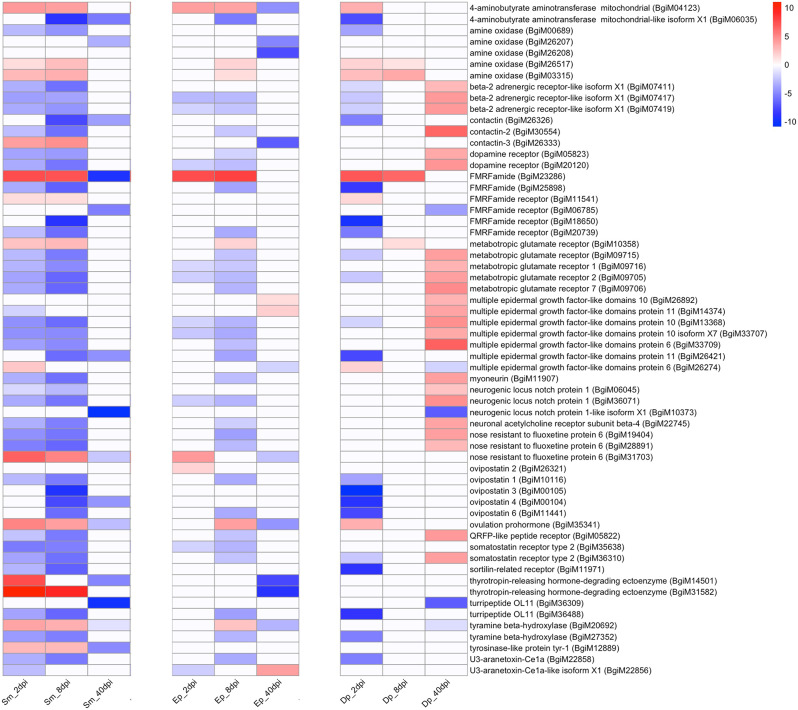
Heat map for genes encoding proteins involved in neural development or maintenance, neuro-endocrine communication and extracellular signaling. Not all variants are shown for each named gene (e.g. 2 of 3 known *FMRFamides* are shown)

We identified 203 genes among our DE transcripts that fell into this broad category (Table S21), of which 60 were of interest based on their altered expression in our three groups of parasite-infected snails (Fig. 8). Noteworthy among them are genes encoding polypeptides that eventually yield cleaved active peptides which are the largest group of extracellular signaling molecules in molluscs [74]. These peptides may be released and act locally on other neural cells (neural peptides) or be released into the hemolymph and affect target sites far from their source (peptide hormones). Both often interact with G protein-coupled receptors to transduce their effects to recipient cells.

One example is *ovulation prohormone* (Fig. 8), a gene homologous to the gene from *Lymnaea stagnalis* known to encode a polypeptide yielding two neuroactive peptides of particular relevance, caudodorsal cell hormone (or CDCH), which induces ovulation in *L. stagnalis*, and calfluxin, which causes an influx of calcium into mitochondria of cells in the albumen gland thereby activating synthesis of the gland’s egg and egg mass fluids [69, 75]. We examined the complete iM line *B. glabrata* ovulation prohormone polypeptide sequence and recovered a homolog for *L. stagnalis* CDCH (BgiM35341-RA with coverage: 59%, identity: 34.97%) but did not find a calfluxin homolog within *ovulation prohormone* or elsewhere in the iM line genome. As might be expected, in unexposed controls, this gene is strongly up-regulated in iM line adults relative to juveniles. Interestingly, relative to unexposed controls, *ovulation prohormone* was up-regulated in *S. mansoni*-exposed snails at 2 and 8dpi and in *E. paraensei*-exposed snails at 8dpi, but then down-regulated at 40dpi in snails infected with either trematode. Schistosomin, described as an antagonist of calfluxin, and a possible mediator of parasitic castration in *L. stagnalis* [75, 76], was not observed to be DE in iM line *B. glabrata* (see also [77, 78]).

Other peptide-encoding genes with complex patterns of expression were *FMRFamides* and *ovipostatin* genes (Fig. 8; see Table S21 for details about all the remaining genes discussed in this section). The receptors for bioactive peptides or bioamine neurotransmitters may also represent key targets for parasite manipulation. Among receptors we found for which expression was altered by parasite infection (Fig. 8) were *beta-2 adrenergic receptor-like isoform X1, dopamine receptor* and *FMRFamide receptor*, *neuronal acetylcholine receptor subunit beta-4*, *QRFP-like peptide receptor*, *somatostatin receptor type 2* and *neurogenic locus notch protein*.

Another way neurohormonal control of reproduction in snails could be disrupted by parasites is by impairing the cleavage of peptides from precursor polypeptides. In addition to the five proteases mentioned in Fig S4 (see also Table S20) found to be strongly down-regulated at 40dpi with *E. paraensei*, *4-aminobutyrate aminotransferase mitochondrial-like isoform X1* and *thyrotropin-releasing hormone-degrading ectoenzyme* (Fig. 8) provide two additional examples.

Enzymes responsible for maintaining levels of neurotransmitter pools are another possible parasite target. The effect of parasites we saw on down-regulating *tyrosinase* activity (Fig. 7) and the limiting effects this might have on levels of dopamine provide one possibility. Two additional possibilities are *amine oxidase* and *tyramine beta-hydroxylase* (8 genes) which are involved in modification of bioamines and exhibit complex responses upon exposure to parasites (Fig. 8).

Yet other possibilities exist to disrupt reproduction including alteration of intercellular transport of nutrients. The gene *nose resistant to fluoxetine protein 6*, for which at least 8 versions exist in *B. glabrata*, 3 of which are shown in Fig. 8, may play a key role in uptake of a range of nutrients, and their down-regulation in infected snails may be indicative of a diversion of nutrients from host to parasite tissues. We also identified 5 groups of genes that may play an important role in nervous system growth, development and viability and that are responsive to infection, one being *multiple epidermal growth factor-like domain* (represented by 51 genes, 7 shown in Fig. 8) which promotes growth of dendrites and overall showed strong down-regulation in parasite-infected snails. The remaining groups include *contactins*, *metabotropic glutamate receptors*, *myoneurin* and *sortilin-related receptor* (Fig. 8; additional details in Table S21).

The snail genome encodes some unusual toxins such as *turripeptide OL11* with similarities to toxins produced by sea slugs, and *U3-aranetoxin-Ce1*, with similarities to arthropod toxins (Fig. 8). They are believed to be ion channel inhibitors and their expression is consistently down-regulated in infected snails. Perhaps their down-regulation enables some ion channels to convey signals enabling pathways responsible for repressing reproduction.

### Additional groups of genes examined for differential expression

We also examined the impact of infection with *S. mansoni*, *E. paraensei* or *D. potomaca* on the transcriptomic responses of iM line *B. glabrata* proteases and protease inhibitors (Fig S4, Table S20) and immune-related genes, including: 1) fibrinogen-related domain-containing proteins or FReDs (Fig S5, Table S22); G-protein coupled receptors or GPCRs (Fig S6, Table S23); biomphalysins (Fig S7, Table S24); lectins (Fig S8, Table S25); and other immune genes including *Toll-like receptors* (TLRs), *thioester-containing genes*, complement-associated genes, *Hemagglutinin/amebocyte aggregation factor, peptidoglycan-binding/recognition, L-selectin*, *macrophage mannose receptor 1*, and *heat shock* genes (Table S26).

### Some comments on non-annotated genes

For each of the three parasites and time points, several iM line *B. glabrata* genes were strongly DE, but lack any annotation (designated NA). Many of these are among the most highly expressed genes as based on read counts, serving to remind us there is much yet to learn about the basic functioning of molluscan physiology.

Even though unannotated, such genes can provide tantalizing insights. For instance, in a list of 893 NA genes up-regulated at 40dpi with *D. potomaca*, 20 were selected that were particularly prominent in having high read count values > 1000 among all replicates (Table S27). They were also all significantly *up*-regulated in the *D. potomaca*-infected snails as compared to unexposed control *adult* snails). We then searched the list of 1135 NA genes in snails infected with *S. mansoni* for 8d for the same genes. Remarkably, all 20 were found and all were also found to be highly expressed in *juvenile* control snails (read counts > 1000), but all were found to be *down*-regulated in snails with 8d *S. mansoni* infections. This pattern is similar to what we have noted in previous sections regarding annotated genes: key genes related to reproduction in snails infected for 8d with *S. mansoni* were down-regulated, but were often up-regulated in snails exposed to *D. potomaca* for 40d.

## Discussion

Using RNA-Seq we aimed to characterize the responses of the model gastropod iM line *B. glabrata* to infection with each of three different metazoan parasites: the digenetic trematodes *S. mansoni* or *E. paraensei*, or the nematode (*D. potomaca*). In each case, the parasite successfully undertakes a complex developmental program that takes weeks to complete, culminating in the production and release of either cercariae in the case of the digeneans, or of infective adult females worms in the case of *D. potomaca*. Whereas the digeneans may continue to steadily produce cercariae for months, infections with *D. potomaca* are essentially self-limiting in that death of the host snail typically follows the emergence of the gravid/inseminated female nematodes (Fig. 1).

We used iM line *B. glabrata* as the host because genome resources available for this highly inbred snail line enable a more complete assembly and deeper level of annotation than achieved with other *Biomphalaria* genomic resources [4, 5, 11]. Furthermore, iM line snails retain a near 100% level of compatibility with *S. mansoni* [4, 5], the best known of all digenetic trematodes, thereby providing an excellent model system for further investigation of mollusc-parasite interactions. Our choice of parasites was further dictated by their availability, ease in lab maintenance, and because collectively they provide a broad understanding of the impacts of parasitism on *B. glabrata*.

As shortcomings, we were able to analyze only three biological replicates for each parasite and time point, and the PCA plot of the various treatments and replicates indicated variability among the replicates. In our experience, variability inevitably occurs in interactions involving *Biomphalaria*-parasite interactions [51, 79] and must be acknowledged as one of the biological realities of such systems. For instance, it is difficult to know exactly where individual snails are in the maturation process and based on our experience, we have used the criteria of specified size ranges and no egg production vs. egg production to delineate juveniles from adult snails, even though some aspects of maturation may have already been initiated in some juveniles. Additionally, the extent of parasite development, as indicated by parasite read counts, is inevitably variable among replicates for a particular parasite and time point. Nonetheless, significant differences in host gene expression can still be ascertained among the 3 parasite species and 3 time points.

Our experimental design can also be criticized for the lack of an unexposed control group snails for the 8d time point because infected snails collected at 8d had six additional days to develop than the 2d controls with which they were compared. However, all snails in the study at the 8d time point were juveniles, and snails with 8d trematode infections were typically *even more* adversely affected with respect to reproduction-related transcripts than snails with 2d infections, suggesting any direct comparison with 8d control snails (also presumably more mature) would only have enhanced the trends we detected using the 2d controls as a baseline. The same line of reasoning argues against the possibility that the effects we saw were simply due to the fact that our 2d control snails had matured prematurely relative to groups of snails exposed to trematodes. If this were so, snails with 8d infections would have shown smaller differences with the 2d controls when in fact the differences were larger, again indicative of an increased trematode effect, also supported by increased parasite read counts (Fig S9) at 8 as compared to 2dpi. Also, there is evidence of specificity in the trematode-mediated effects insofar as dyneins associated with axonemal function (as in the flagella of sperm tails) were down-regulated whereas cytoplasmic dyneins involved in intracellular transport were not (Fig S3).

Another caveat is that there are many unannotated genes in *B. glabrata* that are highly responsive to parasite infection and for which we lack any known homologs or functional understanding. Also, it is conceivable that snail genes that are not strongly DE in our results might still be impactful in host-parasite interactions. As one example, the *leucine aminopetidase 2* gene was identified by Wang et al. [78] as possibly playing a key role in regulating intramolluscan parasite physiology. Although we did not find it to be DE, this by no means excludes it as a key player. We also note that transcript abundance does not automatically equate with translated protein abundance.

With these caveats in mind, each of the three parasites provoked a distinctive host response, but that in many ways the responses evoked by the two trematode species were more similar to one another than either was to the response provoked by the nematode. An excess of down-regulated gene responses was generally recorded for snails with either trematode species, a tendency noted in other studies [20, 26, 51, 78], but not all [79]. This was true both for general overviews (Figs. 3 and 4; Table 3) and for specific groups of genes, particularly those involved in gonado- and gametogenesis (Figs. 5, 6 and 7 and Figs S2, S3 and Tables S14-S18). For all three time points, read counts for *E. paraensei* were lower than for *S. mansoni*, suggesting the former species was slower to develop and fully occupy host tissues, offering a general explanation for why its impacts on host gene expression were less than noted for *S. mansoni*. There were instances, as for egg mass proteins (Fig. 7), for which the expression patterns of *S. mansoni* and *D. potomaca* were more similar, with a puzzling absence of negative effects of *E. paraensei* (the latter discussed further below).

The most surprising result of our study was that as early as 2dpi by *S. mansoni*, and to a lesser extent *E. paraensei*, whole suites of genes associated with gametogenesis were down-regulated, especially those involved in production of sperm, but also some genes believed to be involved in oogenesis. This was supported by a pathway analysis indicating that several key components of signaling pathways controlling meiosis were down-regulated in snails harboring trematode larvae (Fig S2). Genes encoding “yolk proteins” *vitellogenin-4* and *yolk ferritin* produced by the ovotestis and digestive gland, respectively, were also down-regulated. Caution is nonetheless required in interpreting the large impacts of early trematode infection on host reproductive processes. The relative amount of trematode biomass in snails infected for 2 and 8 days is small – only mother sporocysts or mother sporocysts containing developing daughter sporocysts (or mother rediae in the case of *E. paraensei*) would be present.

The wide array of host reproductive genes affected was surprising because we had not observed a comparable effect in previous RNA-Seq studies of outbred juvenile M line snails infected with *S. mansoni* [51]. Our interpretation is that the “juvenile” inbred iM line snails used for the present study had in fact initiated gonadogenesis and gametogenesis, whereas juvenile M line snails used in the previous study had not – that is, M line snails mature more slowly than iM line snails. Fortuitously, our timing was appropriate to see the impact parasite infection had on sexual maturation in its early developmental stages in iM line snails. Experimental infection of juvenile snails with digenetic trematodes typically results in infected snails that have underdeveloped gonadal tissue and rarely if ever produce progeny [71], suggesting that early stages of sexual development are indeed “nipped in the bud”. From these lines of reasoning, we conclude the impacts on gonado/gametogenesis noted most prominently in *S. mansoni*-infected snails are due to the infection and not to starting differences in the extent of host maturation. Others have also noted relatively early onset effects of trematode infection on castration in the 7–12 day post-exposure range [33, 78, 80, 81], or identified snail genes such as *ovipostatin 2* and a *type 1 serotonin receptor* down-regulated at 1dpi that would seem to have adverse effects on reproduction [79].

It is difficult to explain the effects on gametogenesis solely as a result of an excessive energy drain imposed by the trematodes because their biomass and energy demands would be low at 2 and 8dpi. This suggests that castrating trematodes not only consume energy as every parasite does, but also directly modify host reproductive physiology, first shutting down reproduction in line with the “active castrator” concept advanced by Baudoin [82] and Hall et al. [83]. Although *D. potomaca* has some similar tendencies to down-regulate these processes (Figs. 5 and 6; Table 3), it was by no means comparable in scope and magnitude to that imposed by the trematodes, corresponding to the observation that *D. potomaca* does not castrate its snail host as trematodes do [9].

Surprisingly, evidence for comparable effects on gametogenesis in snails with 40d trematode infections was lacking. This may reflect the fact that genes involved are not as active in fully mature control snails or are activated only at certain times such that no demonstrable down-regulation was seen. This is supported by low read counts for these genes noted in adult control snails at that time. By contrast, genes encoding proteins associated with egg or egg mass fluids are among the most highly transcribed genes in adult *B. glabrata*, and many of them were strongly down-regulated in snails with 40d *S. mansoni* or *D. potomaca* infections (Fig. 7).

This suggests that parasitic castration can be imposed by mechanisms other than just attacking gonado- or gametogenesis [81]. So, for mature snails with developed ovotestes at the time of exposure, or that have been able to partially control the proliferation of infection, an alternative or complementary parasite strategy of castration may be to target the energy-demanding processes of egg and egg mass production that are largely facilitated by the female accessory sex organs (albumen, pars contorta, oothecal, and muciparous glands).

*E. paraensei* infections of 40d duration provide yet additional possible modes of imposing castration. In this case, genes in the female accessory genitalia were not strongly down-regulated, but expression of *vitellogenin A2* was, suggesting that ovum-supportive proteins produced by the ovary itself can also be targeted. Additionally, *E. paraensei* had pronounced negative effects on the production of several host proteases that might impair processing of peptides, the latter being stimulatory to egg production. One likely player in reduced egg/egg mass protein production at 40dpi in trematode-infected snails is the diminished expression of the *ovulation prohormone* gene by the caudodorsal cells of the snail’s cerebral ganglion. Among the processed peptides derived from the prohormone polypeptide is the CDCH which stimulates ovulation.

A distinctive role for ovulation prohormone in *Biomphalaria* reproduction is also suggested by the fact that it is upregulated at 2 and 8dpi in trematode-infected juvenile snails, at a time when their ovotestis and accessory reproductive organs are still largely undeveloped. Why this up-regulation might occur is counterintuitive, but it suggests that the snail host can sense the parasite’s presence and its production could represent an attempt to hasten egg production. Infection of snails in which maturity has been attained is sometimes accompanied by a temporary increase in egg production above the level seen in uninfected controls, which is then followed by castration. This phenomenon, called fecundity compensation [84], although not seen in our infected snails, might in other cases be enabled by an early upsurge in ovulation prohormone such as the one noted in juvenile snails.

In our model system, trematodes served as probes to elicit host responses that help to identify for the first time many *Biomphalaria* genes relevant to their reproduction and that are responsive to parasite manipulation. Although studies of genes involved in gonadogenesis and gametogenesis are in their infancy in molluscs, recent studies in abalones and bivalves draw attention to several groups of molecules that seem to be involved in the sexual maturation process [67, 85–88]. Many of the genes identified in these studies were also down-regulated in iM line snails at 2 and 8dpi following exposure to *S. mansoni* and *E. paraensei*. We can thus begin to define sets of snail genes relevant to understanding the widely appreciated but poorly understood process of trematode-mediated castration. We include in Table 4 a selected list of genes that we believe at least in part define targets of trematode-mediated castration in *B. glabrata*, particularly as it might occur in juvenile snails, but also include potential targets for snails exposed to infection when reproductively mature. As these genes are integral to successful reproduction and directly involved in growing and maintaining populations of schistosome vector snails in nature, they could potentially become useful targets for more selective means of snail control.

**Table 4:** A subset of the genes from iM line *B. glabrata* involved in reproductive development, gamete production, and egg or egg mass production implicated as targets of trematode-mediated castration that might serve as useful markers for this process, and as potential targets for control operations

**General sexual development**	
BgiM13571	DMRTI or 1 doublesex- and mab-3-related transcription factor 2
BgiM10972	DNA-directed RNA polymerase II subunit RPB1
BgiM24147	forkhead box protein P1
BgiM03615	FOX or forkhead box protein B1-like isoform X1
BgiM00177	GATA zinc finger domain-containing protein 14
BgiM22838	GATA zinc finger domain-containing protein 15
BgiM22839	GATA zinc finger domain-containing protein 15
BgiM12852	nanos 1-like isoform X2
BgiM26970	piwi-like protein 1
BgiM03226	protein boule-like isoform X2
BgiM14501	thyrotropin-releasing hormone-degrading ectoenzyme
BgiM07364	vasa (ATP-dependent RNA helicase DHX57)
	E3 ubiquitin-protein ligases*
	endothelin converting enzyme 1, 2 and -like 1*
	kelch-like proteins*
	FMRFamides*
	ovipostatins*
	zinc finger proteins*
**Meiosis**	
BgiM04788	meiosis-specific with OB domain-containing protein-like
BgiM02188	meiotic recombination protein REC8-like protein
BgiM12503	meiotic recombination protein SPO11-like isoform X1
**Testis development/spermatogenesis**	
BgiM36176	cilia- and flagella-associated protein 52
BgiM27401	dynein heavy chain 8 axonemal
BgiM31883	G2/mitotic-specific cyclin-B3
BgiM29077	histone H2B.2 sperm-like
BgiM11775	kelch-like protein 10
BgiM12727	nardilysin
BgiM08071	testis-specific serine/threonine-protein kinase 2
BgiM08069	testis-specific serine/threonine-protein kinase 3
BgiM13792	tudor domain-containing protein 1*
**Ovarian development/oogenesis**	
BgiM32459	cell division cycle protein 20
BgiM24484	MAM and LDL-receptor class A domain-containing protein 1-like isoform X2
BgiM24348	MAM and LDL-receptor class A domain-containing protein 2
BgiM12520	vitellogenin-4
	E3 ubiquitin-protein ligases*
**Egg/egg mass production**	
BgiM17528	bactericidal permeability-increasing protein
BgiM17527	LBP/BP1
BgiM17525	LBP/BP1.2
BgiM35341	ovulation prohormone**
BgiM20266	tyrosinase-like protein -tyr-3
BgiM12520	vitellogenin-4
BgiM28031	vitellogenin-A2
BgiM00991	yolk ferritin

*Note:* The genes listed here were notable for the extent and/or persistence of their down-regulation following trematode infection but in some unusual cases might also be up-regulated, possibly as a means to ensure some reproductive success for the snail (forms of fecundity compensation – see text). Assignation of some of these genes to the specific categories listed should be considered provisional, as some of the genes may play roles in several aspects of reproductive physiology and there is much to be clarified with respect to their exact function and specificity

*Indicates general categories for which several different genes might be involved in the response

**This gene might contribute to fecundity compensation early in the course of infection in some trematode-mollusc systems

A final consideration of the impact of parasite infection on *Biomphalaria* reproduction relates to *D. potomaca*, which is totally different in its mode of parasitism from the two trematodes. Particularly in light of the inhibitory effect of *D. potomaca* on snail egg mass protein production at 40d (Fig. 7), it was most surprising that so many genes from such snails were otherwise up-regulated, including many of the genes we associated with gonado- or gametogenesis that were down-regulated at earlier time points in trematode infected snails. The up-regulated responses in 40d *D. potomaca* snails were, with respect to dozens of genes, exactly the opposite response as seen in snails with 2 or 8d trematode infections. This unusual symmetry, apparent even though two separate groups of baseline control snails were involved, helps to better define the nature of the genes used by *B. glabrata* in reproductive development and gametogenesis (Table 4). This result also implies pathways associated with younger snails can also be activated in snails of the age and size of adults and supports the idea that different, but simultaneous, avenues of parasite effects exist, one stimulating gametogenesis and another interfering with activating egg production, both occurring in snails with 40d *D. potomaca* infections.

Furthermore, snails in the terminal stages of infection with *D. potomaca* can sense their deteriorating condition with one potential response being to mount a last-ditch attempt at additional reproduction. The up-regulated gametogenesis response in *D. potomaca*-infected snails could also represent a form of fecundity compensation, although of a somewhat different nature than described above for trematode-infected snails. In the case of *D. potomaca*, it seems an attempt by the host is made to enhance reproductive effort at the terminal stages of parasite development, before it succumbs rather than at an early stage of parasite development, as with trematode-infected snails.

Regarding the involvement of immune/defense genes in response to the three parasites, even though each parasite is able to complete successfully its development in iM line snails, a large variety of proteases, protease inhibitors, FReD genes, biomphalysins, and lectin genes were responsive at each of the different time points. Unlike in other *B. glabrata* strains such as BS-90s (or iBS90) which are capable of mounting aggressive and distinct early resistance responses able to prevent parasite proliferation [51], in the combinations used in the present study, the parasites prevail. In such a situation, particularly at 40d when parasites have heavily colonized snails and are producing and releasing their progeny, the host responses seen may be more to limit internal or integumental damage caused by hundreds of parasites regularly seeking to exit from the host. Also, given that the host’s normal physiology has been radically altered by parasitism, it is conceivable that opportunistic pathogens might colonize the infected host and compromise its survival, as also noted by Schultz et al. [27], requiring a change in defense strategy as the original parasite infection ages. However, at least among the immune components we examined, pronounced up-regulation of putative immune/defense factors was not pronounced at 40dpi.

## Conclusions

Three different compatible metazoan parasites (two trematodes and one nematode) provoked distinctive responses from snails of the inbred iM line *B. glabrata* strain, providing evidence that digenetic trematode larvae begin to interfere with a broad array of newly-identified *Biomphalaria* gonado-and gametogenesis associated host genes as early as 2dpi. We also noted separate suites of genes related to ovulation and egg mass production were down-regulated by 40dpi, including *ovulation prohormone* from which is derived CDCH hormone implicated in triggering ovulation. We provide a list of host genes we believe serve as markers of trematode-induced castration and that might be useful as targets in snail control programs. We identified genes potentially involved in two different forms of fecundity compensation, one involving the nematode *D. potomaca*, and provide additional information on an array of snail defense genes potentially involved in retaining host integrity in the face of overwhelming metazoan infections.

### Electronic supplementary material

Below is the link to the electronic supplementary material.

Supplementary Material 1

Supplementary Material 2

Supplementary Material 3

Supplementary Material 4

Supplementary Material 5

Supplementary Material 6

Supplementary Material 7

Supplementary Material 8

Supplementary Material 9

Supplementary Material 10

Supplementary Material 11

Supplementary Material 12

Supplementary Material 13

Supplementary Material 14

Supplementary Material 15

Supplementary Material 16

Supplementary Material 17

Supplementary Material 18

Supplementary Material 19

Supplementary Material 20

Supplementary Material 21

Supplementary Material 22

Supplementary Material 23

Supplementary Material 24

Supplementary Material 25

Supplementary Material 26

Supplementary Material 27

Supplementary Material 28

Supplementary Material 29

Supplementary Material 30

Supplementary Material 31

Supplementary Material 32

Supplementary Material 33

Supplementary Material 34

Supplementary Material 35

Supplementary Material 36

## Data Availability

The transcriptomic sequencing data of the samples in this study are available at NCBI under project accession: PRJNA892730. All other data generated or analyzed during this study are included in this published article and its supplementary information files.

## Abbreviations

ASW: Artificial spring water
CDCH: Caudodorsal cell hormone
DE: Differential gene expression
dpi: Day post-infection
FBG: Fibrinogen
FC: Fold change
FReDs: Fibrinogen domain-containing proteins
FREPs: Fibrinogen-related proteins
GPCRs: G-protein coupled receptors
IgSF: Immunoglobulin superfamily
KEGG: Kyoto Encyclopedia of Genes and Genomes
PCA: Principal components analysis
PPDE: Posterior probability of differential expression
TLRs: Toll-like receptors
