# Supplementary figures and images for: Different metazoan parasites, different transcriptomic responses, with new insights on parasitic castration by digenetic trematodes in the schistosome vector snail Biomphalaria glabrata
Source: BMC Genomics. 2024 Jun 17;25:608. doi: 10.1186/s12864-024-10454-4 (PMC11184841; doi:10.1186/s12864-024-10454-4)

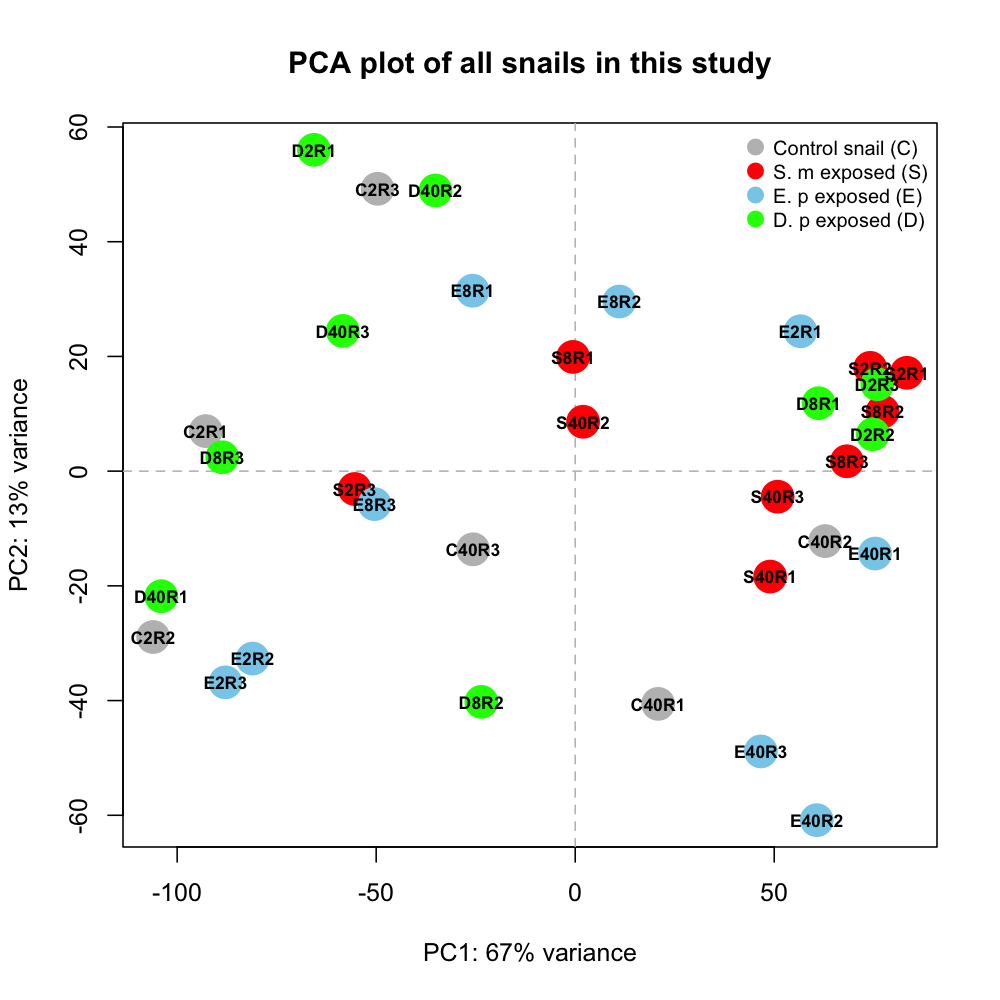

Supplement: Supplementary file 28 — Supplementary Material 28 [file 12864_2024_10454_MOESM28_ESM.jpg]

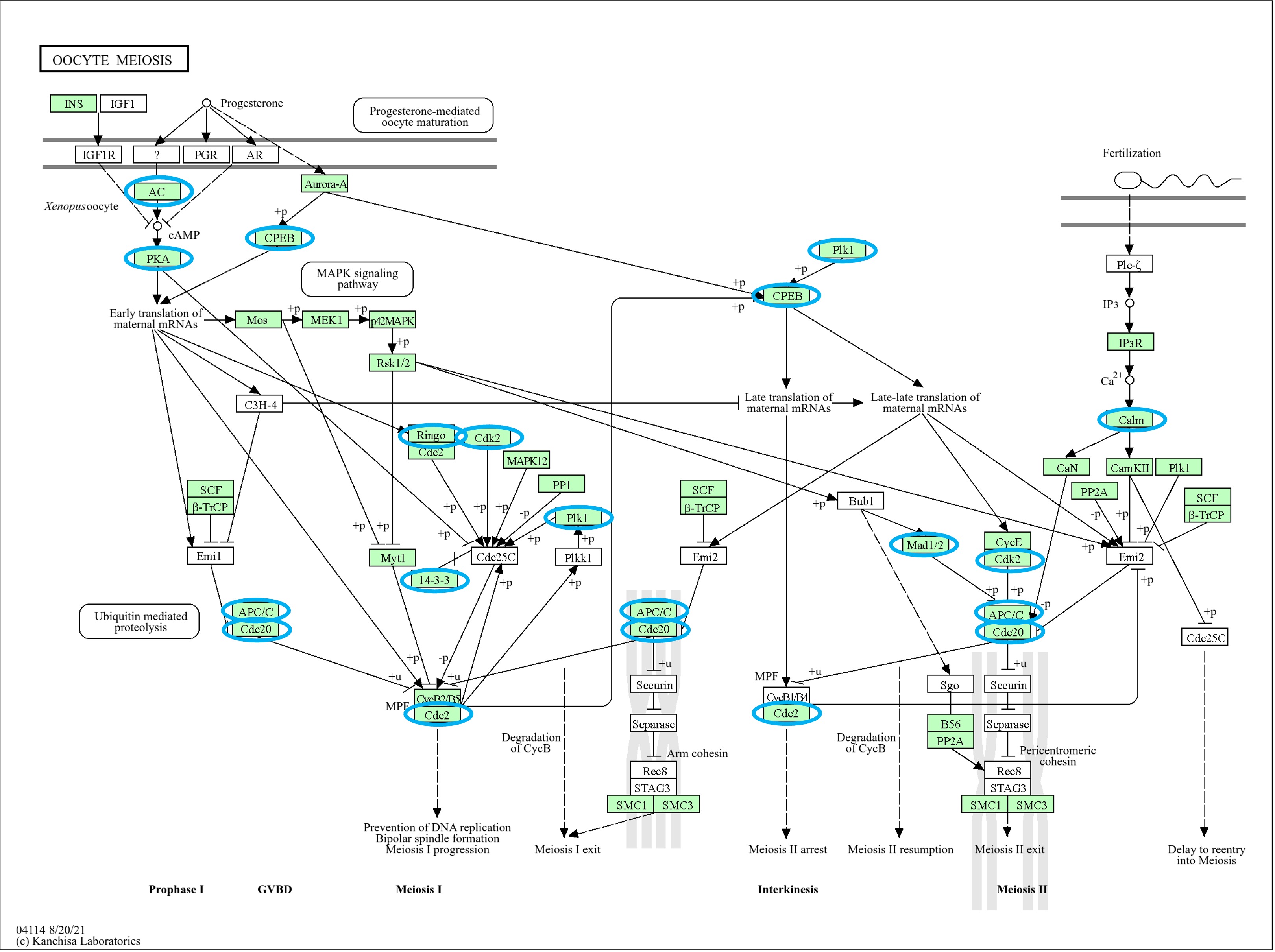

Supplement: Supplementary file 29 — Supplementary Material 29 [file 12864_2024_10454_MOESM29_ESM.jpg]

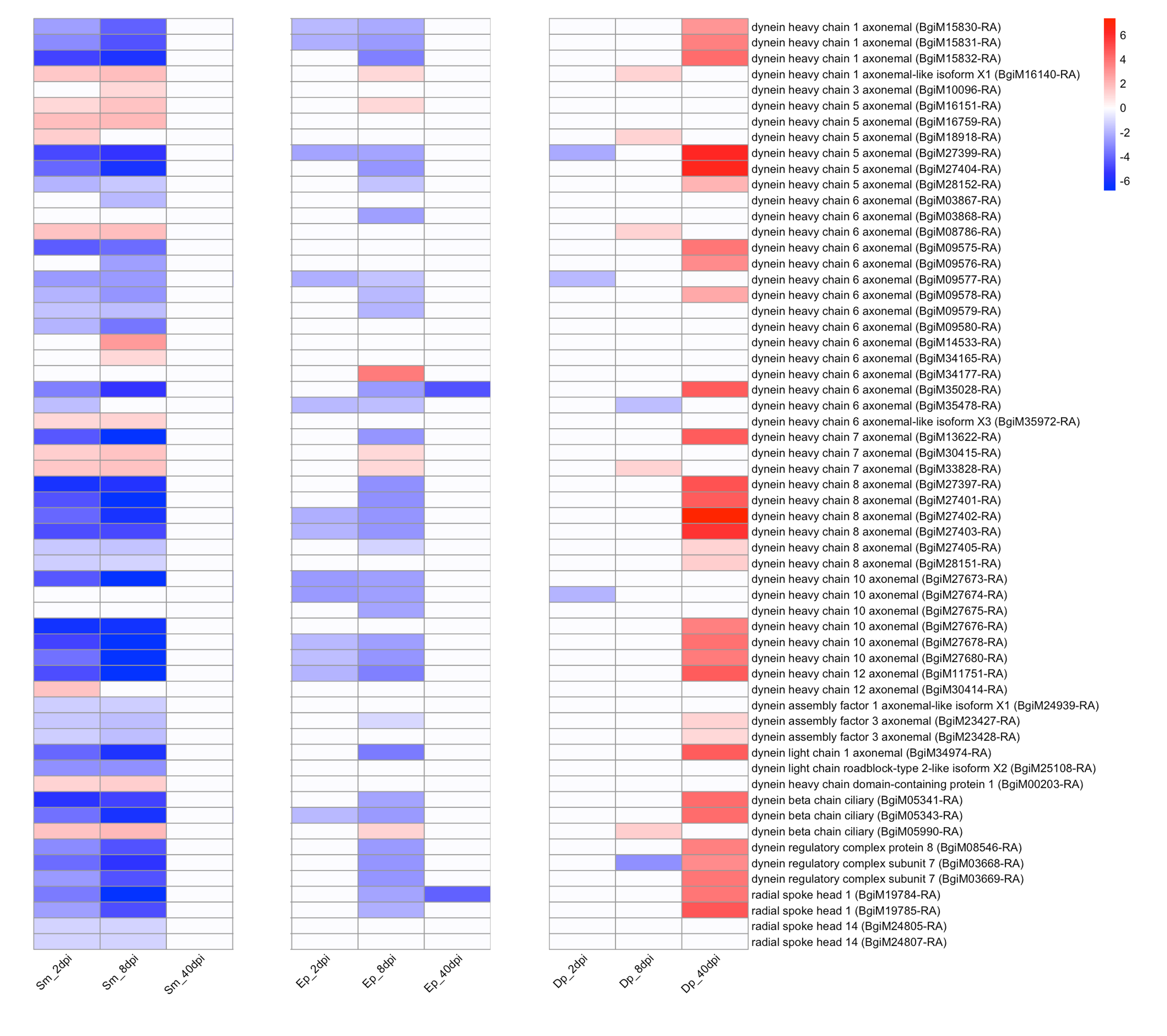

Supplement: Supplementary file 30 — Supplementary Material 30 [file 12864_2024_10454_MOESM30_ESM.jpg]

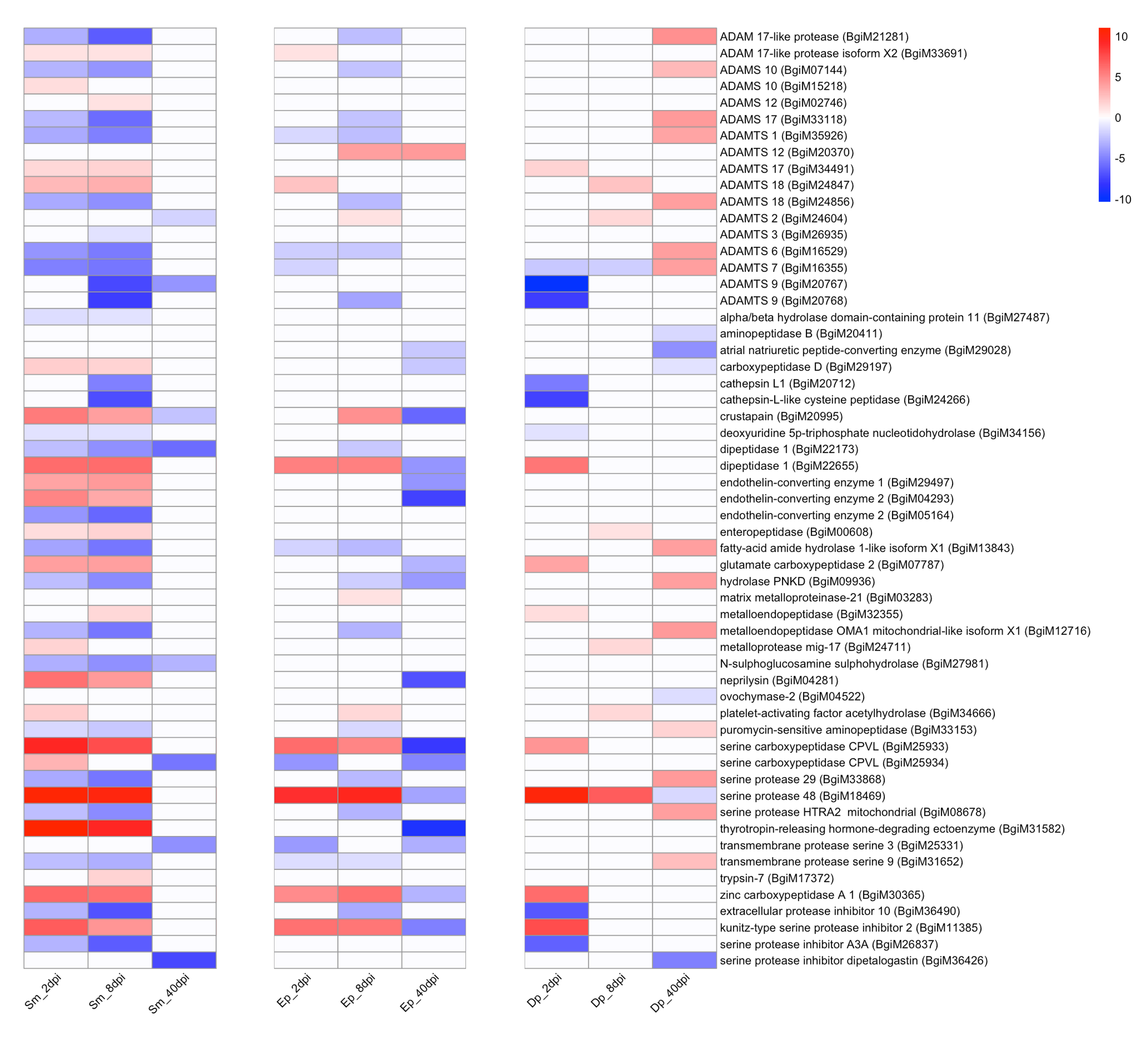

Supplement: Supplementary file 31 — Supplementary Material 31 [file 12864_2024_10454_MOESM31_ESM.jpg]

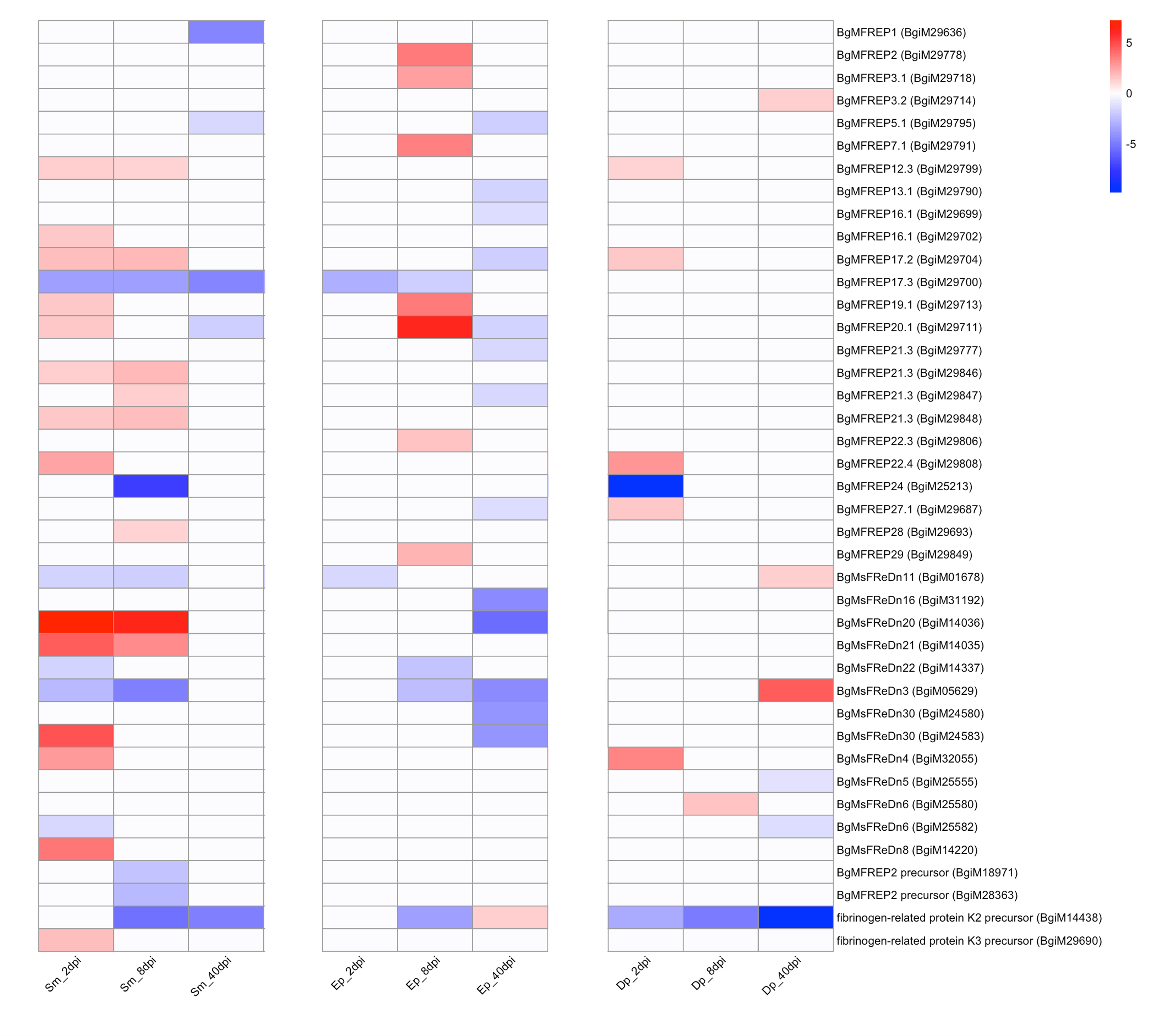

Supplement: Supplementary file 32 — Supplementary Material 32 [file 12864_2024_10454_MOESM32_ESM.jpg]

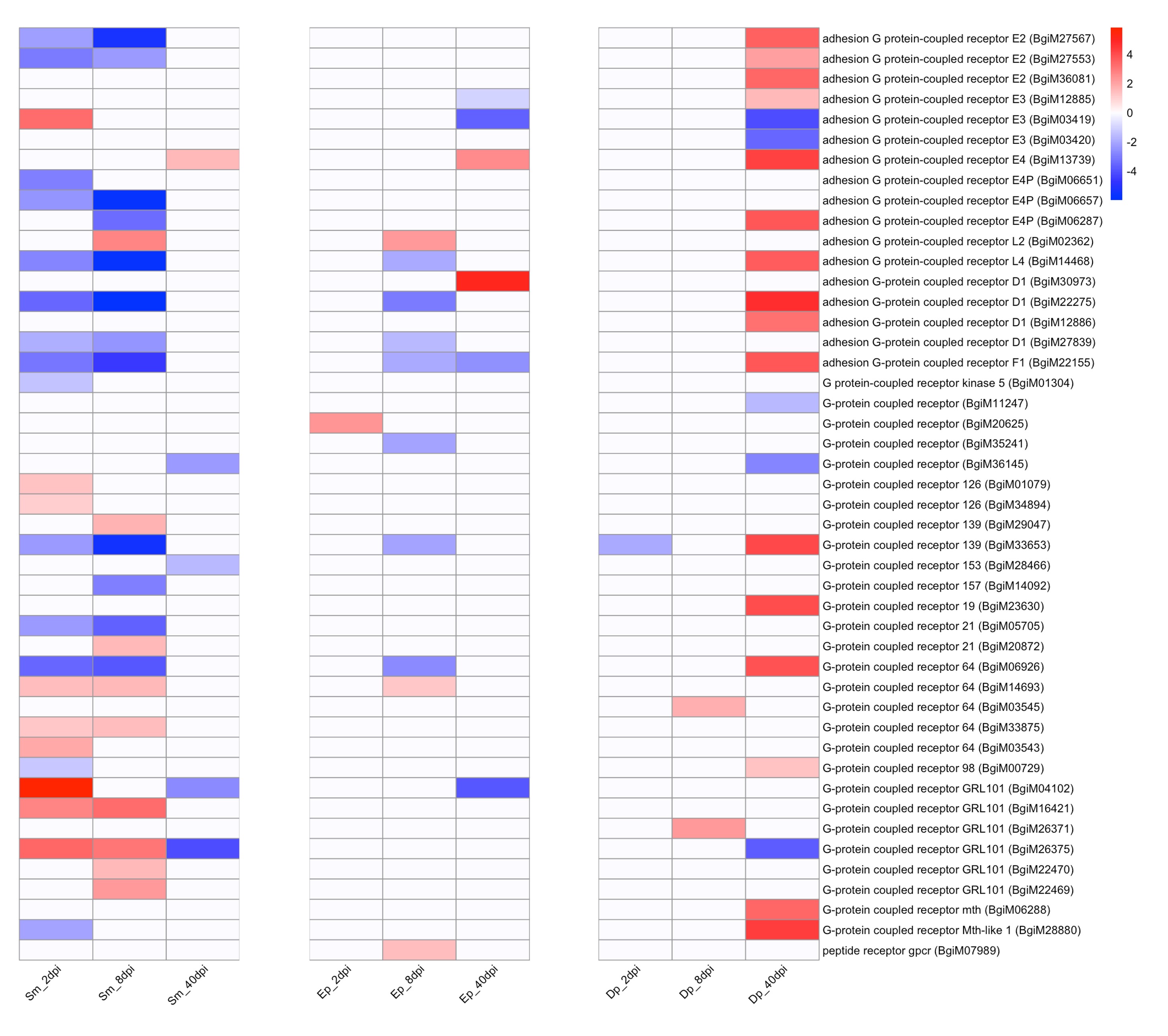

Supplement: Supplementary file 33 — Supplementary Material 33 [file 12864_2024_10454_MOESM33_ESM.jpg]

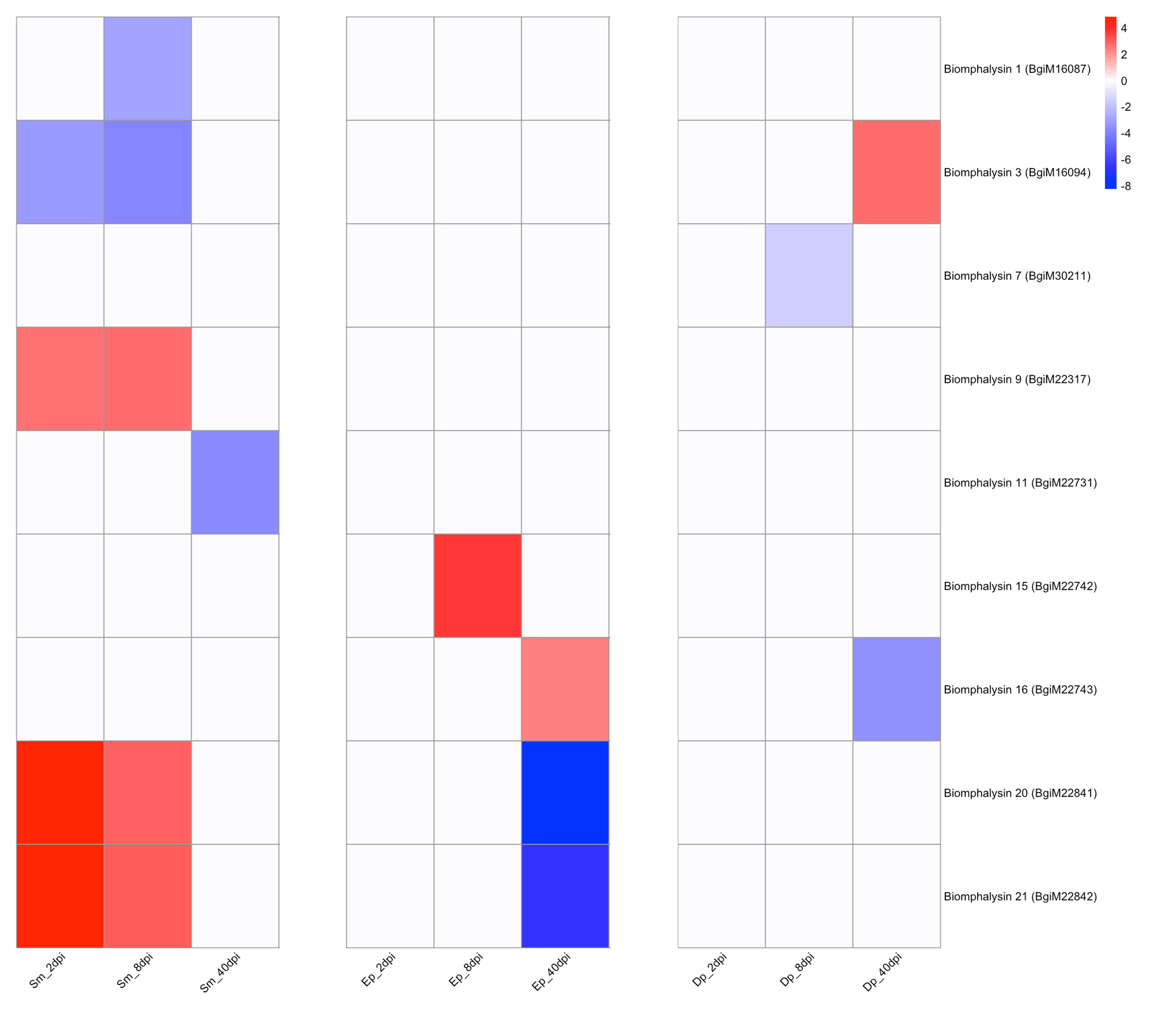

Supplement: Supplementary file 34 — Supplementary Material 34 [file 12864_2024_10454_MOESM34_ESM.jpg]

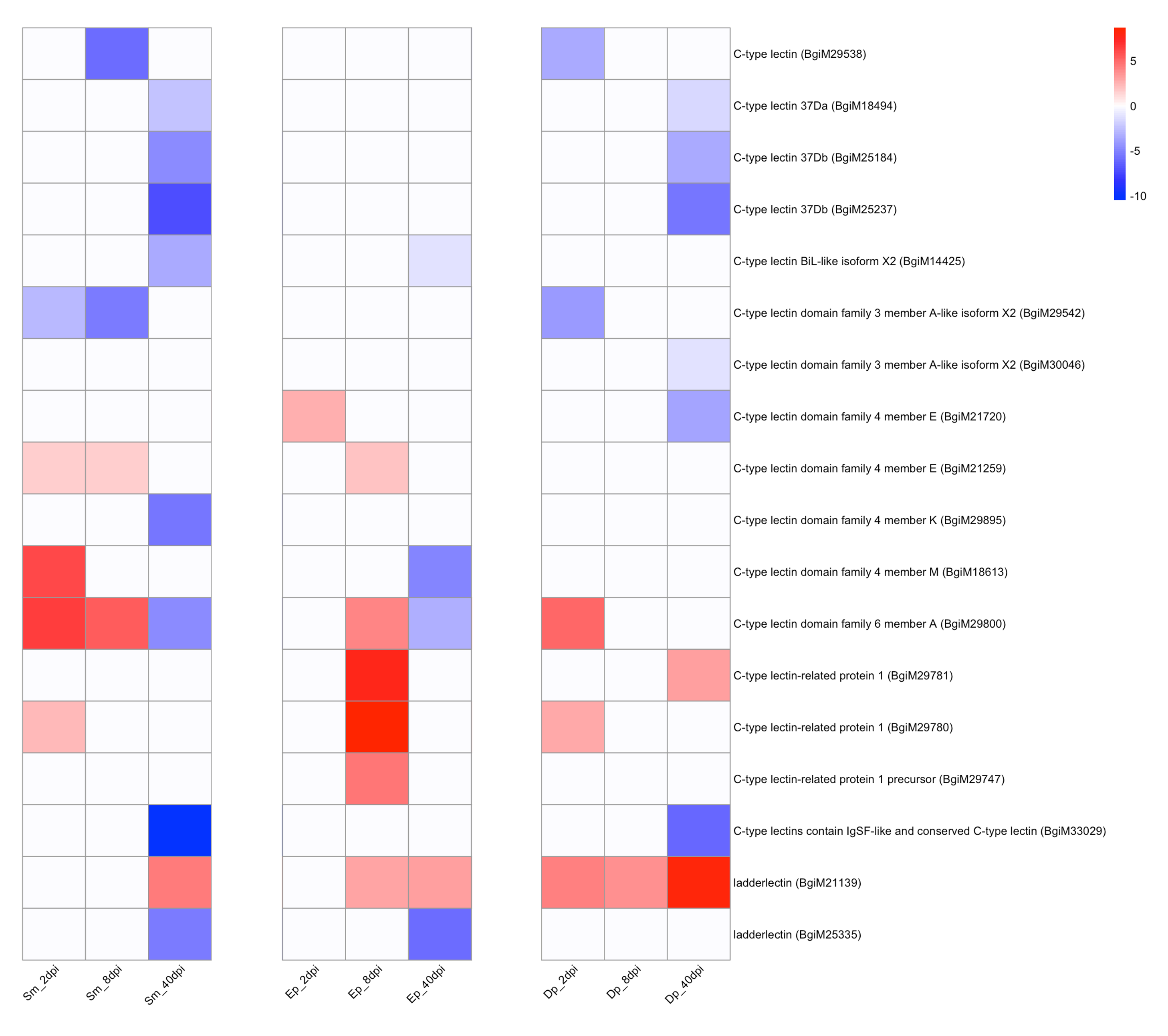

Supplement: Supplementary file 35 — Supplementary Material 35 [file 12864_2024_10454_MOESM35_ESM.jpg]

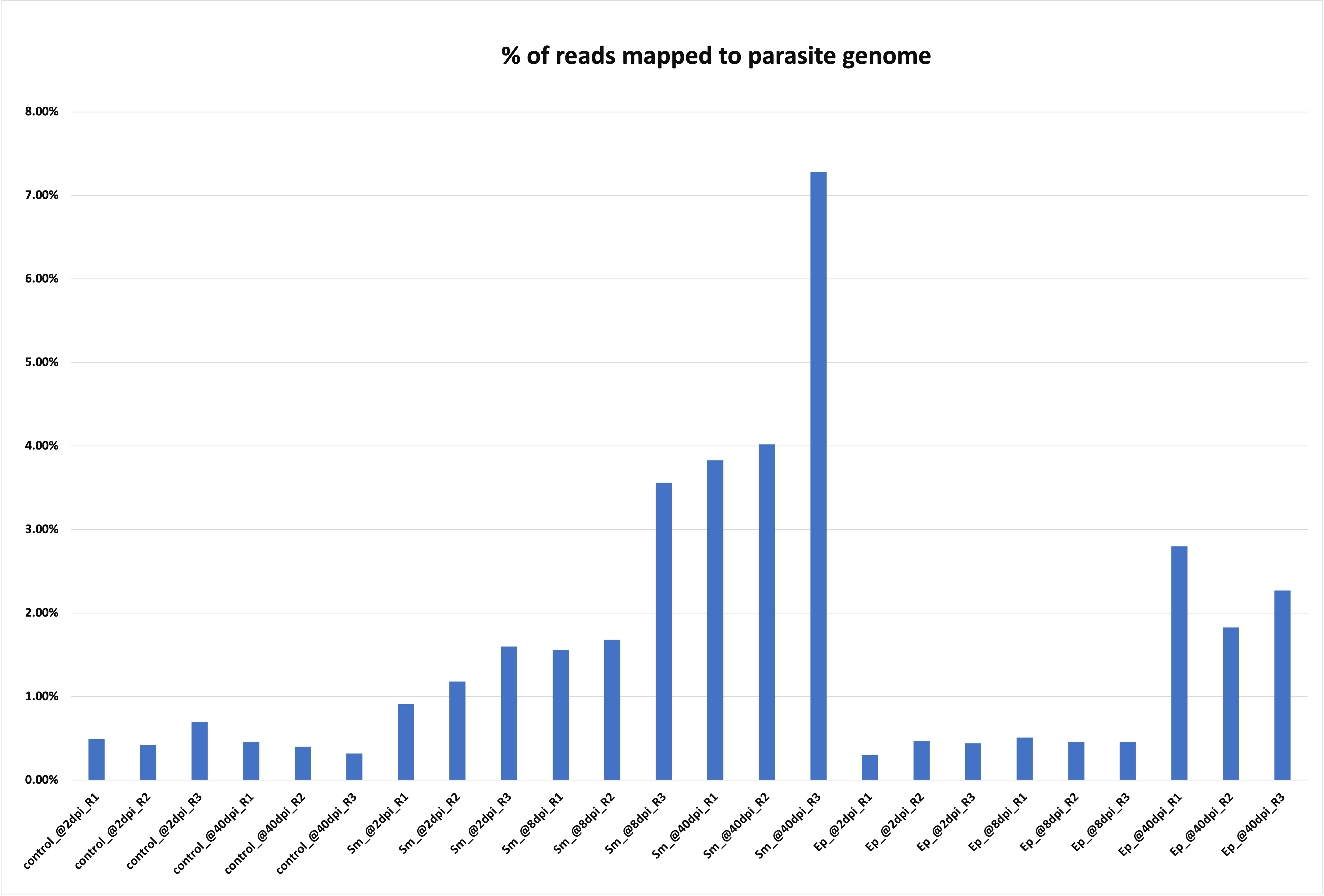

Supplement: Supplementary file 36 — Supplementary Material 36 [file 12864_2024_10454_MOESM36_ESM.jpg]
